# HSPA2 influences the differentiation and production of immunomodulatory mediators in human immortalized epidermal keratinocyte lines

**DOI:** 10.1038/s41419-025-07565-5

**Published:** 2025-04-26

**Authors:** Agnieszka Gogler, Agata Małgorzata Wilk, Damian Robert Sojka, Małgorzata Adamiec-Organiściok, Natalia Matysiak, Daria Kania, Klaudia Wiecha, Ewa Małusecka, Alexander Jorge Cortez, Dawid Zamojski, Michał Marczyk, Agnieszka Maria Mazurek, Sylwia Oziębło, Dorota Scieglinska

**Affiliations:** 1 Center for Translational Research and Molecular Biology of Cancer, Maria Sklodowska-Curie National Research Institute of Oncology Gliwice Branch, Wybrzeze Armii Krajowej 15, 44-102 Gliwice, Poland; 2 Department of Biostatistics and Bioinformatics, Maria Sklodowska-Curie National Research Institute of Oncology Gliwice Branch, Wybrzeze Armii Krajowej 15, 44-102 Gliwice, Poland; 3 Department of Systems Biology and Engineering, Silesian University of Technology, Akademicka 16, 44-100 Gliwice, Poland; 4 Department of Histology and Cell Pathology, Faculty of Medical Sciences in Zabrze, Medical University of Silesia in Katowice, Jordana 19, 41-808 Zabrze, Poland; 5 Department of Data Science and Engineering, Silesian University of Technology, Akademicka 16, 44-100 Gliwice, Poland; 6 Genetic Laboratory, Gyncentrum Sp. z o.o., 41-208 Sosnowiec, Poland; 7 Yale Cancer Center, Yale School of Medicine, New Haven, CT USA

**Keywords:** Differentiation, Transcriptomics

## Abstract

Chaperone proteins constitute a molecular machinery that controls proteostasis. HSPA2 is a heat shock-non-inducible member of the human HSPA/HSP70 family, which includes several highly homologous chaperone proteins. HSPA2 exhibits a cell type-specific expression pattern in the testis, brain, and multilayered epithelia. It is a crucial male fertility-related factor, but its role in somatic cells is poorly understood. Previously, we found that HSPA2 deficiency can impair epidermal keratinocyte differentiation. In this study, we confirmed the crucial role of HSPA2 in keratinocyte differentiation by investigating immortalized keratinocytes cultured in a reconstructed human epidermis model. Moreover, we uncovered the influence of HSPA2 on immunomodulation. Transcriptomic analysis revealed that the total loss of HSPA2 affected the expression of genes related to keratinocyte differentiation and interleukin- and interferon-mediated signaling. The functional analysis confirmed bidirectional changes associated with the loss of HSPA2. The HSPA2 knockout in HaCaT and Ker-CT keratinocytes, but not HSPA2 overproduction, impaired granular layer development as evidenced by reduced levels of late keratinocyte differentiation markers, filaggrin and involucrin, along with structural abnormalities in the upper epidermal layer. Differentiation defects were accompanied by increased mRNA expression and extracellular secretion of keratinocyte-derived pro-inflammatory IL-6 cytokine and CCL2, CCL8, CXCL1, CXCL6, and CXCL10 chemokines. The loss of HSPA2 also led to increased expression of extracellular HSPA1 and interferon-stimulated genes and secretion of immune cell modulator SLAMF7. Knocking down HSPA1 expression in keratinocytes decreased the secretion of IL-6 and CCL5 release, suggesting extracellular HSPA1’s role in the HSPA2-regulated molecular network. To summarize, we uncovered the complex homeostatic role of HSPA2 in epidermal keratinocytes. Our results suggest that dysfunction in HSPA2 activity could be an important pathogenicity factor and potential therapeutic target for inflammatory cutaneous diseases.

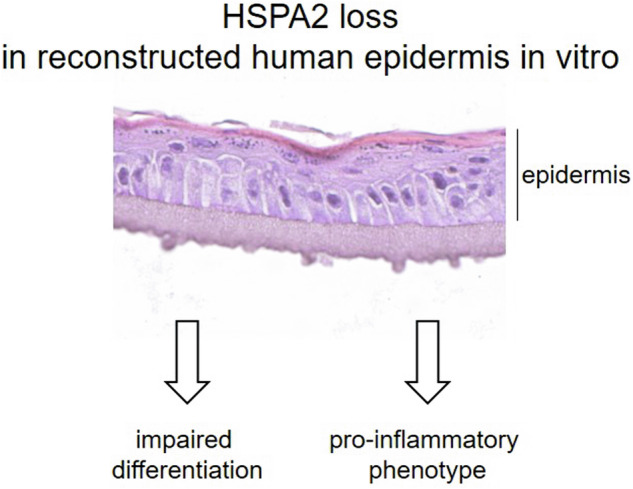

## Introduction

Chaperone proteins are essential for maintaining cellular proteostasis. The human HSPA (HSP70) family comprises twelve chaperones that assist in folding newly synthesized or misfolded proteins and in targeting irreversibly damaged proteins to degradation [1]. HSPAs are one of the most evolutionary conserved proteins. The amino acid sequence similarity of the five most homologous paralogs (HSPA1, HSPA1L, HSPA2, HSPA6, and HSPA8) exceeds eighty percent [2]. These HSPAs share the same subcellular localization shuttling between the cytoplasm and nucleus [3, 4], and can be released extracellularly as soluble proteins and within small extracellular vesicle cargo [5–8]. The extent to which these homologous HSPAs overlap functionally is still unclear. HSPAs, which have many binding partners [9], can impact numerous signaling pathways. The influence of HSPAs on cellular signaling has been intensively studied in cancer models, where redundant action of HSPA paralogs confers potent pro-cancerous effects [10–12]. However, the outcome of single- or double-paralog-specific HSPA inactivation may vary, depending on the molecular background of the cancer cells and the specific paralog under study [10, 13, 14]. The significance of HSPA paralogs in human non-cancerous cells is much less understood.

It is believed that in humans and other complex organisms, the major differences among HSPAs arise from their distinct expression patterns. Except for HSPA8, a housekeeping chaperone in virtually all cell types, the basal levels of HSPA paralogs can vary depending on the physiology of the cell and tissue [15, 16]. Majority of HSPAs are encoded by stress-inducible genes, whose expression increases due to activation of the so-called heat shock response, an adaptive mechanism driven by the HSF-1 transcription factor. The heat-shock-non-inducible *HSPA2* gene is an exception because its cell- and tissue-specific expression is regulated in an HSF-1-independent manner [2, 15]. In humans, high levels of HSPA2 are present in spermatogenic cells [17], basal cells of stratified epithelia, including the epidermis, as well as in certain other organs [15–18]. However, stem cells in the seminiferous epithelium [19] or epidermis do not express HSPA2 [18].

HSPA2 is essential for sperm differentiation and function [20], but its role in extratesticular cells is not well understood [2]. We have recently shown that downregulation of the *HSPA2* gene in immortalized human keratinocytes affects their clonogenic potential and the expression of differentiation markers, indicating a defect in keratinocyte differentiation [18]. This finding was intriguing, given that the function of HSPAs in the epidermis has been rather associated with protection against environmental stresses, so far [21, 22].

This study aimed to assess the effect of HSPA2 on the keratinocyte phenotype. We used the spontaneously immortalized epidermal human keratinocyte lines, considered an alternative to primary human epidermal keratinocytes (HEK). HaCaT cells retain the basic characteristics of HEK, including the ability to differentiate and orchestrate inflammatory responses [23]. This choice allowed us to circumvent the limitations of donor-to-donor variability inherent in primary HEKs. Such variability could hinder the detection of potentially subtle functional changes induced by HSPA2 loss. Additionally, it helped avoid issues related to the intrinsic resistance of HEKs to CRISPR/Cas9-mediated gene editing [24]. Using the RNA-Seq approach, we identified which signaling pathways are most sensitive to HSPA2 activity in reconstructed human epidermis (RHE) in vitro. By employing HSPA2-knockout (KO) immortalized keratinocyte lines, we found a complex role for HSPA2 in maintaining epidermal homeostasis. This chaperone regulates keratinocyte differentiation and ensures the proper extracellular release of immunomodulatory mediators.

## Materials and methods

### Cell lines and culture conditions

HaCaT cell line was purchased from CLS GmbH (RRID: CVCL_0038, Eppelheim, Germany; authenticated in 2023) and cultured in DMEM (glucose 4.5 g/l; Merck KGaA, Darmstadt, Germany) supplemented with 10% heat-inactivated FBS (EURx, Gdansk, Poland) and gentamycin. BJ1-hTERT, hTERT-immortalized human skin fibroblast line (RRID: CVCL_6573), and Ker-CT, hTERT- and CDK4-immortalized human primary foreskin keratinocyte line (RRID: CVCL_S877) were purchased from ATCC (Manassas, VA, USA). BJ1-hTERT cells were grown in DMEM/199 medium [3:1] with 0.01 mg/ml hygromycin B and 10% FBS. Ker-CT cells were cultured on collagen-IV-coated plates in CnT-Prime Epithelial Culture Medium (CELLnTEC Advanced Cell Systems AG Bern, Switzerland). Cells were tested for mycoplasma contamination.

### Generation of genetically modified keratinocyte lines

#### HSPA2 overproduction (OVER) model

The construction of the lentiviral vector encoding the HSPA2 protein and the generation of HSPA2+ (control, pLXV) and HSPA2-overproducing (HSPA2-OVER, pLVX-A2) cells were described in [25].

#### HSPA2 knockdown (KD) model

The lentiviral vectors encoding the non-targeting and HSPA2-targeting shRNAs used for the generation of HSPA2+ (control, CTR-luc), and HSPA2- (A2-sh4) cell lines are described in [18].

#### HSPA2 knockout (KO) model

Control HSPA2+ (CRISPR-CTR) and HSPA2- (CRISPR-4, CRISPR-2A, CRISPR-2B, CRISPR-2 and CRISPR-5) cells were generated as described in [26]. CRISPR-CTR cells are a pool of isogenic HSPA2+ clones with the non-edited *HSPA2* gene sequence (six for HaCaT, four for Ker-CT). Pools of isogenic HSPA2- clones with a complete HSPA2 protein loss are as follows: HaCaT – CRISPR-4 (four clones); CRISPR-2A and CRISPR-2B (two distinct clones each); Ker-CT – CRISPR-5 (five clones), CRISPR-2 (two clones).

#### Double HSPA2-/HSPA1- model

CRISPR-4 cells were transduced with lentiviral vectors encoding the control non-targeting and HSPA1-targeting shRNAs [12] to generate control HSPA2-/HSPA1+(Luc), and HSPA2-/HSPA1-(S) and -(N) cells, respectively.

### Reconstructed human epidermis model (RHE)

BJ1-hTERT fibroblasts were used to create fibroblast-seeded collagen gel layer on Corning Transwell® insert with 0.4 μm pores. RHE was generated as described in ref. [18]. HaCaT or Ker-CT cells were seeded on inserts, cultured submerged for 3 days, then grown at the air-liquid interface (ALI) for 12 or 18 days (details in the Supplementary Material and Methods).

Code for quantitative analysis of IHC-stained RHEs is available on GitHub (https://github.com/DawZam/EpidermaQuant) [27].

### RNA-seq data analysis

Sequence reads were subjected to quality assessment and pre-processing (adapter trimming, mapping to reference genome, normalization, and low abundance filtering). Principal component analysis (PCA) was used to identify the main variability sources among various sample subgroups, and top contributing genes for components related to HSPA2 deficiency were subjected to over-representation analysis to identify affected biological processes. Next, supervised analysis and gene set enrichment were performed. Bioinformatic methodology is described in details in Supplementary Material and Methods.

### Statistical analysis

All data are presented as means ± standard deviation (SD). To analyse differences between the groups, a two-tailed Student’s *t* test for two group comparisons was used. *P* value = 0.05 was taken as the threshold for statistical significance.

### Other methods

Other methods, including RNA isolation, RT-PCR (nucleotide sequences of PCR starters are listed in Supplementary Table S1), RNA-Seq, histomorphometric and ultrastructural analysis, immunohistochemistry (IHC), protein extraction, Western blot (WB) analysis (antibodies are listed in Supplementary Table S2), proinflammatory stimulation, and collection of conditioned medium (CM), can be found in the Supplementary Material and Methods. Uncropped WB images are shown in Supplementary Materials.

## Results

### Gene expression profiling and signaling pathways related to the loss of HSPA2

This study was conducted using HaCaT cell variants with HSPA2 knockdown (KD) [18], HSPA2 knockout (KO) [26], and HSPA2 overproduction (OVER) [25] (Supplementary Fig. S1A, B). The HSPA2- cells in KD (A2-sh4) and KO (CRISPR-2A, CRISPR-4) models exhibited partial and complete loss of the HSPA2 protein, respectively, without a compensatory increase in the levels of other HSPA paralogs (Supplementary Fig. S1A).

The influence of HSPA2 on cellular signaling networks was examined by the RNA-Seq method to evaluate transcriptome profiles in the 18-day-old 3D reconstructed human epidermis (RHE) formed by wild-type (wt), KO, and KD cells. The RHE model is considered superior to standard 2D keratinocyte culture as it allows the formation of stratified, epidermis-like tissue in vitro, mimicking spatiotemporal changes associated with keratinocyte differentiation.

**Fig. 1:**
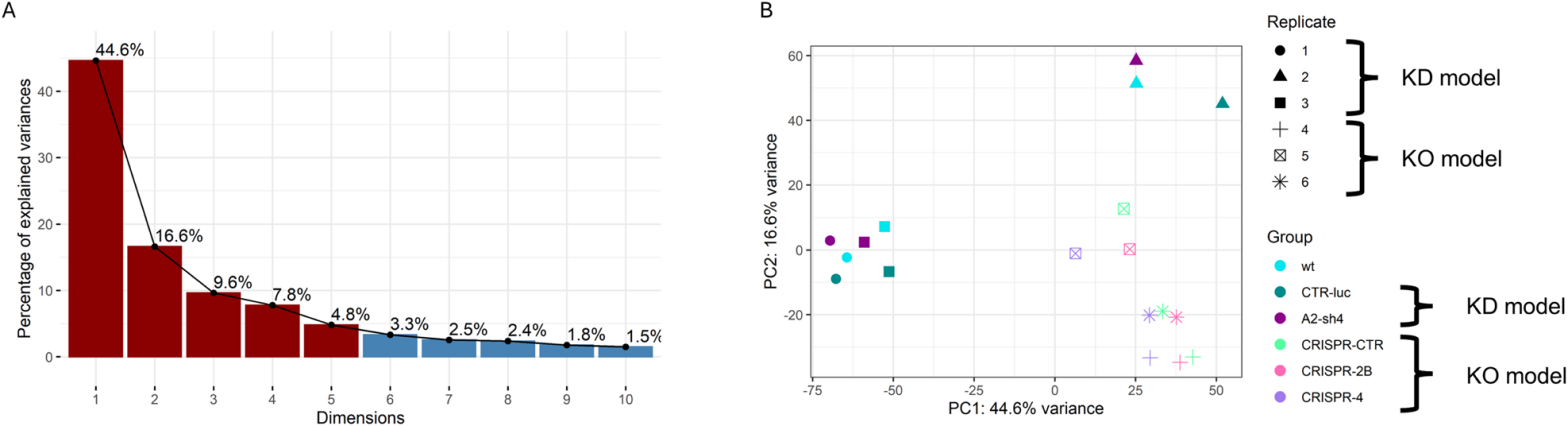
Transcriptomic comparison of the reconstructed human epidermis (RHE) cultures formed by HSPA2+ and HSPA2- HaCaT cells (Principal Component Analysis, PCA). Datasets from KD (knockdown) and KO (knockout) cell models were analyzed together. **A** The percentage of variance explained by consecutive principal components (PC) of rlog-transformed, unscaled expression levels of all genes. Dark red indicates components that collectively explain 80% of the total variance. **B** Two-dimensional scatter plot of scores for the first two PCs. Colors represent different cell variants, while shapes denote replicates. In the KD model, HSPA2- RHE is represented by the A2-sh4 cells (group; purple), while HSPA2+ by the CTR-luc cell variant (dark green); in the KO model, HSPA2- RHE is represented by CRISPR-2B (pink) and CRISPR-4 (violet) cells, while HSPA2+ by CRISPR-CTR (neon green) cells. Wild-type cells (wt, blue) were considered an additional variant of HSPA2+ cells. The remaining PCs are shown in Supplementary Fig. S2A–C. Results of the supervised analysis of HSPA2- *versus* HSPA2 + RHE variants can be found in Supplementary Fig. S2D, E (Supplementary Results).

Using unsupervised Principal Component Analysis (PCA), we identified factors related to major sources of variability in our dataset. For all RHE variants (wt, KO, and KD models), the first principal components (PC) (Fig. 1A) revealed distinct grouping of the samples according to biological replicate and model type (Fig. 1B, Supplementary Fig. S2A). None of the PCs separated the HSPA2+ and HSPA2- RHEs (Fig. 1B, Supplementary Fig. S2A). Despite no clear indication of technical issues related to RNA isolation or sequencing, the inter-replicate variability observed in the KD model was much greater than that between the KO samples, suggesting matters intrinsic to conditions during cell growth in RHE [28].

**Fig. 2:**
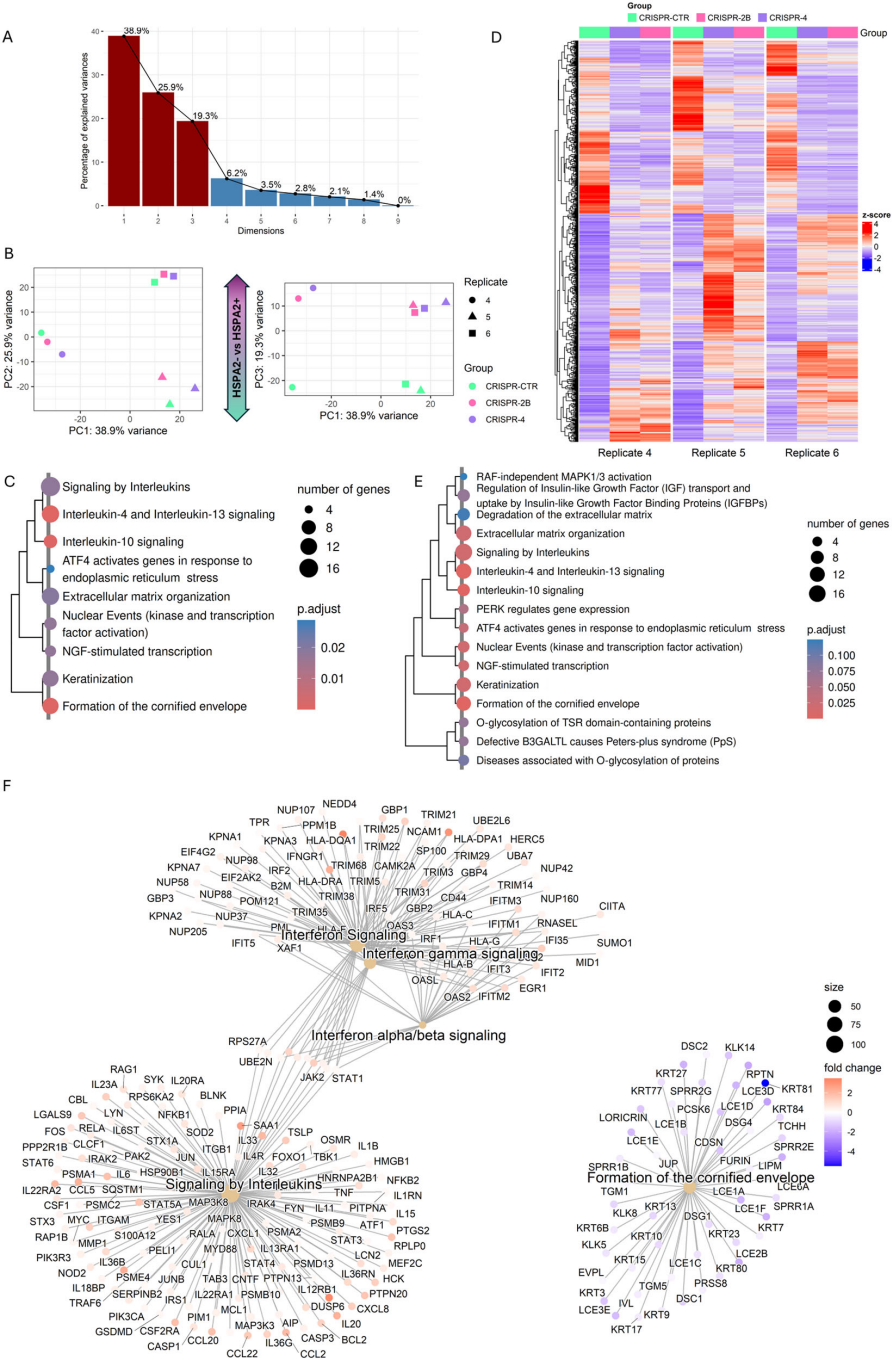
Transcriptomic comparison of the reconstructed human epidermis (RHE) generated by HSPA2+ and HSPA2- cells (the KO model). **A** The percentage of variance explained by consecutive principal components (PC) in a principal component analysis (PCA) of rlog-transformed, unscaled expression levels of all genes. Dark red indicates PCs that explain 80% of the total variance. **B** Two-dimensional scatter plot of scores for the first three PC from the PCA. Colors represent cell modification variants, and shapes represent replicates. HSPA2- RHEs are represented by two variants (groups) of modified cells: CRISPR-2B (pink) and CRISPR-4 (violet), while HSPA2+ by control variant CRISPR-CTR (mint). **C** Significant pathways identified in overrepresentation analysis (ORA) performed on a set of genes extracted from PC3, clustered by term similarity. **D** Heatmap showing expression patterns for differentially expressed genes with |log2FoldChange|>1. For most genes, the direction of expression change in HSPA2- versus HSPA2+ cells was consistent across replicates. **E** The top 20 significant pathways in gene set enrichment analysis (GSEA), clustered by similarity. **F** An annotated gene-concept network for the top 5 enriched pathways from the GSEA analysis. Fold changes for core enrichment-contributing genes in HSPA2- *versus* HSPA2+ RHEs are indicated with colors, with upregulated genes shown in red and downregulated in blue. Expression patterns of genes in the top three enriched pathways are illustrated in Supplementary Fig. S4.

The separate analysis of transcriptomic data from the KD model revealed that none of the PCs, which cumulatively explained 80% of the observed variance, could distinguish between the HSPA2+ and HSPA2- RHEs (details in Supplementary Fig. S3A, B). Therefore, the KD model was excluded from further analysis. In the KO model, although the main source of variability was still associated with biological replicate (Fig. 2A, B), we identified PC3 as being responsible for the HSPA2- versus HSPA2+ difference (Fig. 2B). Over-representation analysis (ORA) performed on the set of top contributing genes extracted from PC3 identified terms related to interleukin signaling and keratinocyte differentiation as significantly altered (Fig. 2C). A substantial number (3507 genes, Padj <0.05) of differentially expressed genes (DEG) showed consistent changes between HSPA2+ and HSPA2- RHEs (Fig. 2D). Gene Set Enrichment Analysis (GSEA) revealed that pathways related to immunomodulation (upregulated) and keratinocyte differentiation (downregulated) were among the most significantly enriched in HSPA2- RHE (Fig. 2E, F).

**Fig. 3:**
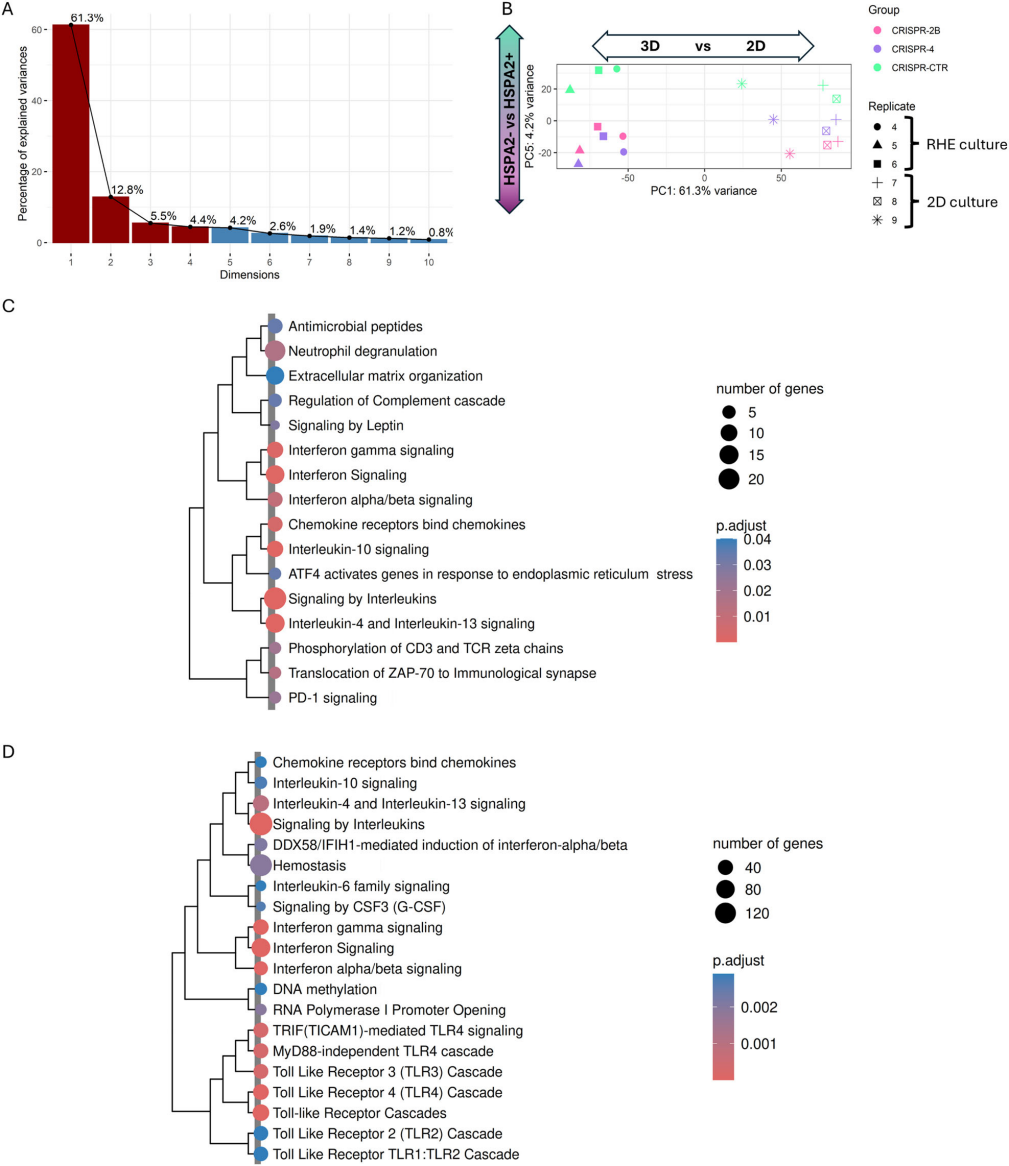
Transcriptomic comparison of HSPA2- *versus* HSPA2+ cells grown in the reconstructed human epidermis (RHE; replicates 4–6) or standard submerged 2D culture (replicates 7–9). **A** The percentage of variance explained by consecutive principal components (PC) for principal component analysis (PCA) of rlog-transformed, unscaled expression levels of all genes. Dark red indicates those components that collectively explain 80% of the total variance. **B** The first and fifth PC from the PCA (with the fifth PC associated with the HSPA2- group). Colors represent cells (groups), and shapes denote replicates. HSPA2- RHEs are represented by two groups of modified cells: CRISPR-2B (pink) and CRISPR-4 (violet), while HSPA2+ by control CRISPR-CTR (mint) cells. The remaining PC are shown in Supplementary Fig. S6A. **C** Significant pathways identified in overrepresentation analysis (ORA) performed on genes selected from PC5. **D** The top 20 significant pathways from gene set enrichment analysis (GSEA) are clustered by similarity. The gene-concept network showing the top 5 enriched pathways in HSPA2- cells is included in Supplementary Fig. S6B.

Next, we examined whether the differences in gene expression related to HSPA2 loss in the KO model are influenced by the spatial organization of cells in RHE. PCA performed on data from cells grown in standard 2D culture revealed that no PC could differentiate between HSPA2+ and HSPA2- samples (Supplementary Fig. S5A, B; more results in Supplementary Fig. S5C, D). Combined analysis of RHE and 2D culture data revealed that PC5 distinguished samples based on HSPA2 expression status (Fig. 3A, B). The over-represented signaling pathways (16 in total), in the set of genes extracted from PC5, were associated with immunomodulation (Fig. 3C). Results of GSEA indicated that pathways related to interleukin and interferon signaling, and Toll-Like Receptors (TLR) cascade were enriched in HSPA2- cells, irrespective of the cell culture type (Supplementary Fig. S6B). Notably, pathways related to keratinocyte differentiation were not found in combined analysis of 2D and RHE cultures.

The RNA-Seq analysis results indicate that gene expression changes associated with the loss of HSPA2 were detectable only in the KO model. In RHE cultures, downregulation of signaling pathways related to keratinocyte differentiation was observed, while both RHE and 2D cultures showed upregulation of pathways associated with immunomodulation. Therefore, the KO model was used for functional analysis of HSPA2 effects on keratinocyte differentiation and immunomodulatory signaling in RHE.

### HSPA2 loss affects keratinocyte differentiation in RHE culture

**Fig. 4:**
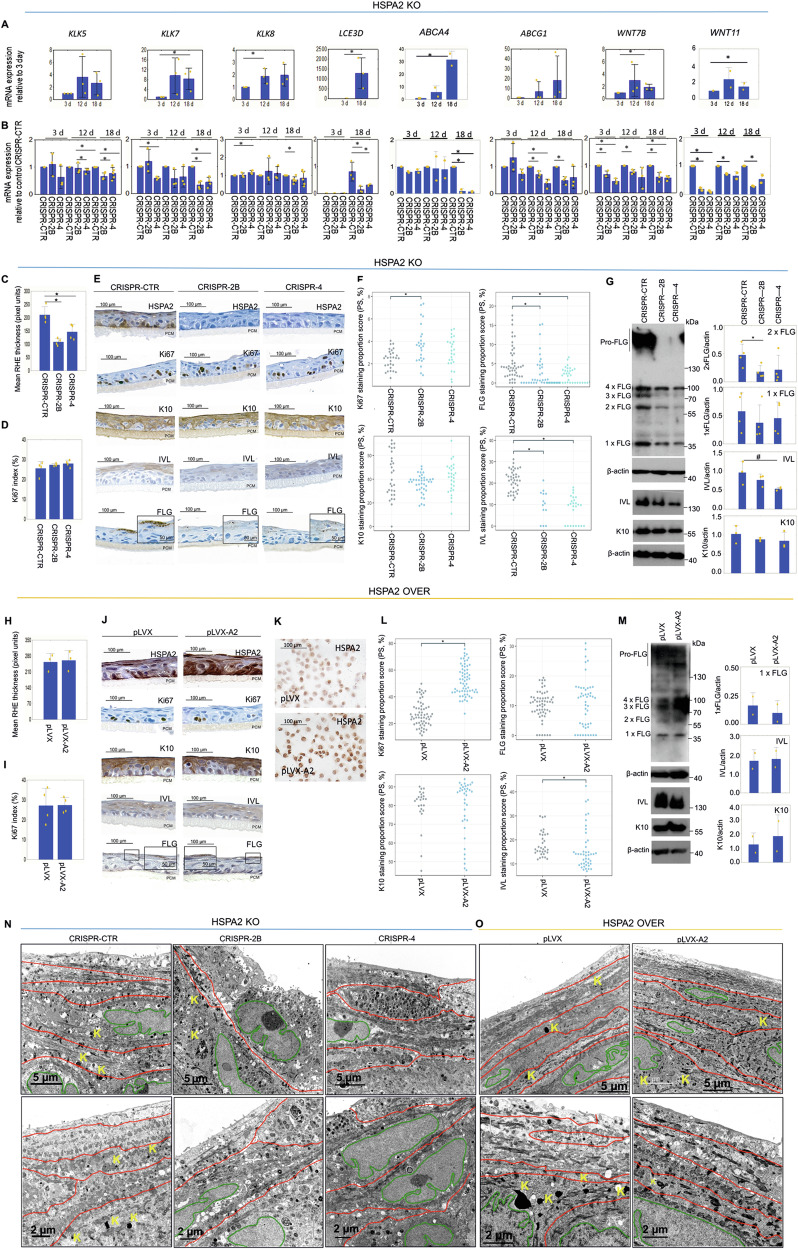
Relationship between HSPA2 levels and HaCaT cell differentiation in reconstructed human epidermis (RHE) cultures. **A** Expression of genes related to keratinocyte differentiation in HSPA2+ (CRISPR-CTR) RHE cultured for 3, 12, and 18 days. Results of RT-qPCR analysis (mean ± SD, *n* = 3 or *n* = 2 in case of *WNT11* and *ABCA4*), each in three technical replicates); gene expression data were normalized to the geometric mean of two reference genes (*TMEM*, *TBCB*). Graphs show fold changes (ΔΔCT) for 18-day RHE, relative to expression in 3-day RHE cultures. **B** Results of RT-qPCR show the expression of differentiation-related genes during the formation of HSPA2+ and HSPA2- (CRISPR-2B, CRISPR-4) RHE cultures at 3, 12, or 18 days. Plots show fold changes (mean ± SD, *n* = 3 or *n* = 2 in the case of *WNT11* and *ABCA4*) calculated relative to HSPA2+ samples (for *LCE3D* relative to reference gene index (ΔCT)). PCR starters are listed in Supplementary Table S1. **C**, **H** Plots show the results of RHE thickness evaluation (mean ± SD) in KO (**C**) and OVER (**H**) models. Hematoxylin-eosin-stained cross-sections of 18-day (fully-developed) RHE samples were measured (KO model, *n* = 4; OVER model, *n* = 3) using ImageJ software. For each biological repeat, at least 6 photomicrographs were analyzed, and the average of 12 measurements of each RHE was used for the statistical analysis. Results are reported in pixel density values. **D**, **I** The Ki-67 proliferation index is calculated as the ratio of Ki-67-positive nuclei to the total number of cells in the basal layer of 18-day RHE. Data are expressed as mean ± SD (*n* = 3). **E**, **J** Representative microphotographs showing DAB-mediated immunostaining of HSPA2, epidermal differentiation markers (K10, FLG, IVL) and proliferation marker (Ki67), in formalin-fixed cross-sections of 18-day RHE. KO model (*n* = 4) (**E**), OVER model (*n* = 5) (**J**). The bar represents 100 µm. **F**, **L** Quantitative analysis of keratinocyte differentiation marker immunostaining, performed using a custom computational algorithm [27]. Each point in the plots represents an analyzed image and corresponds to the result of staining proportion score assessment (KO model, *n* = 4; OVER model, *n* = 3). **G**, **M** Western blot analysis of FLG, IVL, K10 levels in 18-day HSPA2- (**G**) and HSPA2-OVER (**M**) RHEs (*n* = 2). Representative immunoblots are shown. β-actin was used as a protein loading control, with blots generated using 25–35 µg of total protein. **K** Immunocytochemistry-mediated detection of HSPA2 in control and HSPA-OVER cells growing in standard 2D culture (*n* = 2). Antibodies are listed in Supplementary Table S2. **N**, **O** Structural features of the uppermost layers of 18-day RHE cultures formed by cells of KO (**N**) and OVER (**O**) models. Representative microphotographs were taken with transmission electron microscopy. Each red line indicates the cell layer, the green line encircles the nucleus, and the yellow letter K denotes keratohyalin granules. The scale bar represents magnification. The experiment was performed in duplicate. Microphotographs without markings are provided in Supplementary Fig. S7. Statistical significance of differences in (**A**–**D**, **G**, **H**, **M**) was calculated using a two-tailed *t*-test, *P ≤ 0.05. For statistical analysis in (**F**) and (**L**) the Kruskal–Wallis test was used with the post-hoc Nemenyi test to determine significant differences between groups, *P ≤ 0.05.

To validate transcriptomic changes associated with the loss of HSPA2, we used RT-qPCR to examine the expression of core enriched genes in RHE cultures at different growth stages: day 18 (fully developed), day 12, and day 3 (early ALI differentiation). We focused on DEGs annotated in canonical *Keratinization* pathways that are preferentially expressed in the granular layer of the epidermis (*KLK5, KLK7, KLK8. LCE3D*), and on genes linked to the maintenance of epidermal homeostasis through functional studies (*WNT7B, WNT11, ABCA4, ABCG1*) [29–32]. The mRNA levels of the analyzed genes increased with RHE maturation (Fig. 4A), but were lower in HSPA2- compared to HSPA2+ RHE (Fig. 4B). This result confirmed that the loss of HSPA2 impairs the expression of genes related to keratinocyte differentiation.

Parallel microscopic analysis of 18-day-old RHE revealed a multilayered structure composed of basal, spinous, and flattened granular cells, but lacking corneocytes (Fig. 4E, J), consistent with previous report that HaCaT cells are unable to develop the cornified envelope [33]. Importantly, HSPA2- RHEs were significantly thinner than the controls (Fig. 4C). IHC labeling of Ki67 showed that both HSPA2+ and HSPA2- RHEs had similar numbers and localization of proliferating Ki67-positive cells (Fig. 4D, E).

In RHE, we analyzed the expression of HSPA2, as well as early (keratin 10; K10) and late (involucrin, IVL; filaggrin, FLG) keratinocyte differentiation markers by IHC (Fig. 4E). As expected, HSPA2-positive cells were located in the basal layer only in HSPA2+ RHE, reflecting a pattern typical for the epidermis in situ. K10 staining was typical and irrespective of the HSPA2 expression status, but IVL and FLG staining were reduced in HSPA2- RHE (Fig. 4E). Quantitative evaluation of the immunostaining, performed using a self-designed algorithm [27], revealed that K10 staining covered similar regions in both HSPA2+ and HSPA2- RHEs, while the areas showing FLG and IVL staining were smaller in HSPA2- RHE (Fig. 4F). WB analysis confirmed reduced levels of IVL, as well as FLG monomer, dimer, and profilaggrin (FLG precursor) in HSPA2- RHE (Fig. 4G).

Overexpression (OVER model) of HSPA2 did not affect RHE development. Both control and HSPA2-OVER RHEs exhibited similar thickness (Fig. 4H), as well as numbers and distribution of Ki67-positive cells (Fig. 4I, J). Additionally, no differences were observed in the expression of keratinocyte differentiation markers (Fig. 4J, L, M). Compared to the control, HSPA2 staining covered larger areas in HSPA2-OVER RHE (Fig. 4L), but in both cases, HSPA2-positive cells were localized preferentially in the basal layer (Fig. 4J). This observation is noteworthy, as the HSPA2-coding transgene (driven by the constitutive CMV promoter) was homogenously expressed in HSPA2-OVER cells in standard 2D culture (Fig. 4K). This suggests the possibility of transcription-independent mechanisms that might inhibit HSPA2 expression during keratinocyte differentiation.

We analyzed the structure of RHE formed by KO and OVER model cells using transmission electron microscopy. The uppermost layers of HSPA2+ (Fig. 4N) and HSPA2-OVER (pLVX-A2) (Fig. 4O) RHEs were composed of two to four layers of flattened cells without nuclei. In contrast, the uppermost layers of HSPA2- RHEs exhibited structural abnormalities: reduction in the number of flattened cell layers, numerous round-shaped, nuclei-containing cells, rare keratohyalin granules (FLG-containing structures) (Fig. 4N). In summary, our results indicate that the loss of HSPA2 led to the underdevelopment of the granular layer in RHE.

**Fig. 5:**
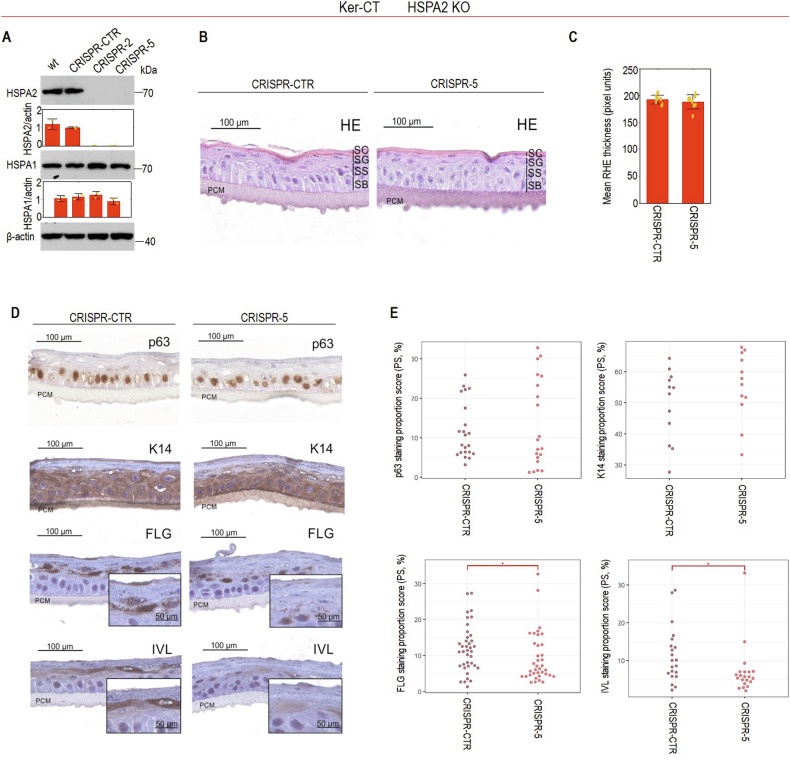
Effects of HSPA2 loss on Ker-CT cell differentiation in reconstructed human epidermis (RHE) cultures. **A** Western Blot detection of HSPA2 and HSPA1 in wild-type (wt), CRISPR/Cas9-edited control (CRISPR-CTR, a pool of four non-edited isogenic clones) and HSPA2-null (CRISPR-2 and CRISPR-5, a pool of two and five HSPA2- isogenic clones, respectively) cells. Representative immunoblots are shown (*n* = 2); β-actin was used as a protein loading control. Graphs below immunoblots show results of densitometry quantification of band intensity. **B** Representative microphotographs showing hematoxylin-eosin-stained cross-sections of 18-day (fully-developed) RHE cultures formed by control and HSPA2- Ker-CT cells, SB – stratum basale, SS – stratum spinosum, SG – stratum granulosum, SC – stratum corneum. **C** Plot shows the results of RHE thickness evaluation (mean ± SD) (*n* = 3; for each biological repeat at least 2 technical RHE samples were analyzed, and the average of 7 measurements of each RHE was used for the statistical analysis) using ImageJ software. Results are reported in pixel density values. **D** Representative microphotographs showing DAB-mediated immunodetection of undifferentiated keratinocyte markers (K14, p63) and epidermal differentiation markers (FLG, IVL) in formalin-fixed cross-sections of 18-day RHE (*n* = 3). The bar represents 100 µm. **E** Quantitative analysis of marker immunostaining. A custom computational algorithm was used [27]. Each point in the plots represents an analyzed image and corresponds to the result of the staining proportion score assessment (*n* = 3). Statistical significance of differences in (**A**, **C**) was calculated using a two-tailed *t*-test, *P ≤ 0.05. For statistical analysis in (**E**) the Kruskal–Wallis test was used with the post-hoc Nemenyi test to determine significant differences between groups, *P ≤ 0.05.

To verify and enhance the translatability of our findings on the impact of HSPA2 loss on keratinocyte differentiation, we generated the HSPA2 KO model in Ker-CT cells. These cells retain basal keratinocyte characteristics, undergo terminal differentiation under high Ca^2+^ stimulation (calcium switch) in the RHE model [34, 35], and have recently emerged as a valuable alternative to primary keratinocytes providing a more physiologically relevant model [36–39]. HSPA2- cells (CRISPR-2, CRISPR-5) showed complete loss of HSPA2 without changes in HSPA1 levels, a key stress-inducible chaperone (Fig. 5A). Upon Ca^2+^-induced differentiation in the RHE model, both HSPA2+ and HSPA2- cells formed multilayered structure with a stratum corneum (Fig. 5B), and similar thickness (Fig. 5C). IHC analysis revealed typical expression of undifferentiated keratinocyte markers (keratin 14; K14; p63), regardless of HSPA2 status (Fig. 5D). However, HSPA2- RHE showed reduced IVL and FLG staining (Fig. 5D), confirmed by quantitative analysis (Fig. 5E). These results indicate that the loss of HSPA2 disrupts late stages of keratinocyte differentiation.

### HSPA2 loss promotes the secretion of immunomodulators by keratinocytes

**Fig. 6:**
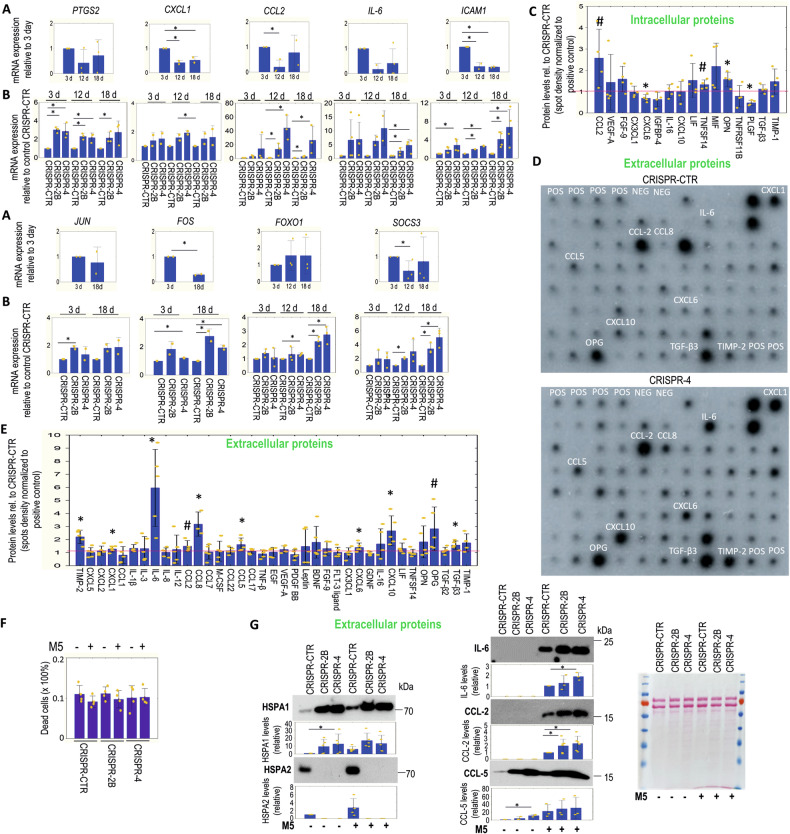
Link between HSPA2 expression levels and the production or secretion of proinflammatory mediators in RHE cultures. **A** Expression of genes involved in *Signaling by Interleukins* pathway in control CRISPR-CTR cells grown for 3, 12, and 18 days in RHE culture. RT-qPCR analysis results (mean ± SD, *n* = 3, or *n* = 2 in case of *CXCL1*, *JUN,* and *FOS*; each with three technical replicates) are shown; gene expression data were normalized to the geometric mean of two reference genes (*TMEM*, *TBCB*). The graph shows fold changes (ΔΔCT) relative to the expression level in the 3-day RHE culture. **B** Expression of genes in *Signaling by Interleukins* pathways during the growth (3, 12, 18 days) of HSPA2+ (CRISPR-CTR) and HSPA2- (CRISPR-2B, CRISPR-4) RHE cultures. RT-qPCR results show fold changes (mean ± SD, *n* = 3, or *n* = 2 in case of *CXCL1*, *JUN* and *FOS*) relative to HSPA2 + RHE cultures. PCR primers are listed in Supplementary Table S1. **C** Intracellular and **D**, **E** extracellular (in conditioned media) levels of pro-inflammatory proteins detected by antibody array in HSPA2+ and HSPA2- RHE cultures after 18 days of growth. The graphs in (**C**) and (**E**) show results (mean ± SD) of densitometric quantification of antibody microarray spots, the data represent the combined measurements for CRISPR-2B and CRISPR-4 RHEs (*n* = 2 for each variant in (**C**), *n* = 3 for each variant in (**E**)) expressed relatively to HSPA2+ RHE after normalization to the internal positive control spots (POS; spots of intensity ≤0.3 relative to POS were excluded from the analysis). In (**D**), representative antibody arrays showing levels of proinflammatory proteins in conditioned medium from HSPA2+ and HSPA2- RHE cultures. **F** Fraction of dead cells detected by trypan blue staining method after 24 h exposure to the M5 cocktail under standard 2D in vitro culture conditions (10 nM concentration of each cytokine). **G** Representative immunoblots (*n* = 3) showing the levels of HSPA1, HSPA2, IL-6, CCL2, and CCL5 proteins in conditioned media from unstimulated and M5-stimulated cells. Graphs below immunoblots show results of densitometry quantification of band intensity. Antibodies used in WB are listed in Supplementary Table S2. Ponceau red-stained blot shows protein loading. 20 µl of concentrated medium was loaded per well. Conditioned media (in **D**, **G**) were collected from cells grown in serum-free OptiMem medium. Statistical significance was calculated using a two-tailed *t*-test, *P ≤ 0.05. In (**C**) and (**E**) *P ≤ 0.05; #*P* ≤ 0.10.

Results from the transcriptome analysis indicated that genes in the *Signaling by Interleukins* pathway were upregulated in HSPA2- RHE (Figs. 2 and 3). To validate this finding, we used RT-qPCR to examine the expression of several core enriched genes (*SOCS3*, *PTGS2*, *CCL2*, *FOXO1*, *CXCL1*, *IL-6*, *ICAM-1*, *JUN*, *FOS*) during RHE differentiation. Except for two genes (*PTGS2*, *FOXO1*), the expression of all other genes decreased in fully-developed RHE (Fig. 6A). All validated genes showed higher expression in more developed HSPA2- RHE compared to HSPA2+ RHE (Fig. 6B), indicating that the loss of HSPA2 is associated with the upregulation of immunomodulation-related genes in mature RHE.

Keratinocytes act as skin sentinels and innate immune cells due to their ability to produce and secrete a wide range of immunomodulatory mediators [40]. Using antibody arrays, we examined the intracellular and extracellular levels of pro-inflammatory mediators in mature RHE (18-day). Among the intracellular proteins, the levels of CCL2, OPN, and TNFS14 differed between HSPA2- and HSPA2+ RHEs (Fig. 6C). In contrast, HSPA2- RHEs released higher levels of several proinflammatory chemokines (CXCL1, CXCL6, CXCL10, CCL2, CCL5, CCL8), cytokines (IL-6, TGF-β3, OPG) and metalloproteinase inhibitor TIMP-2 (Fig. 6D, E).

Next, we used WB to analyze the extracellular levels of CCL2, CCL5, HSPA2, and HSPA1 (eHSPA1), which is considered a damage-associated molecular pattern (DAMP) particle in immunomodulation [41]. In this context, the role of HSPA2 is not well understood [8]. We examined cells grown in 2D culture without and with pro-inflammatory stimulation (24 h) using the M5 cytokine cocktail at a non-toxic concentration (Fig. 6F). The levels of eHSPA1, as well as CCL5 chemokine, were higher in conditioned medium from non-treated HSPA2- cells compared to HSPA2+ cells (Fig. 6G). M5-treated cells released higher levels of HSPA2, IL-6, CCL2, and CCL5, with HSPA2- cells secreting higher levels of IL-6, CCL2 and CCL5 than HSPA2+ cells (Fig. 6G).

**Fig. 7:**
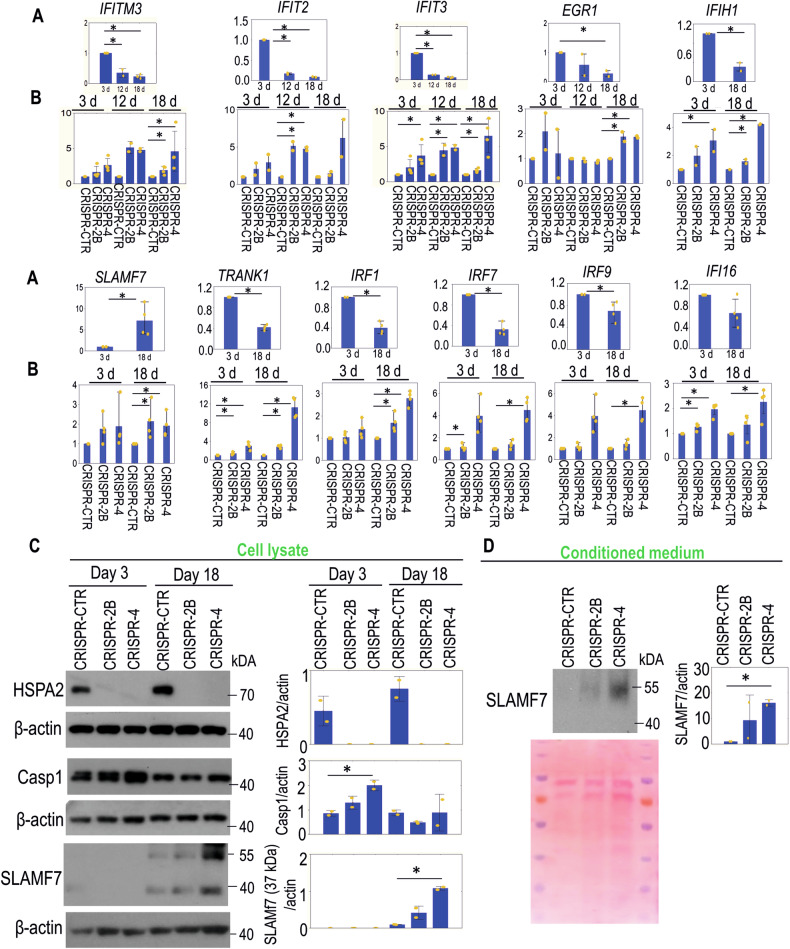
Relationship between HSPA2 expression levels and interferon signaling pathway in HaCaT cells. **A** Expression of genes involved in the *Interferon Signaling* pathway in control (HSPA2 + ) CRISPR-CTR cells grown for 3, 12 and/or 18 days in RHE culture. Results of the RT-qPCR analysis (mean ± SD, *n* = 2 or *n* = 4, each with three technical replicates). Gene expression data were normalized to the geometric mean of two reference genes (*TMEM*, *TBCB*). The graph shows fold changes (ΔΔCT) relative to expression level in 3-day RHE culture. **B** Expression of genes in *Interferon Signaling* pathways during growth (3, 12 and/or 18 days) of HSPA2+ (CRISPR-CTR) and HSPA2- (CRISPR-2B, CRISPR-4) RHE cultures. RT-qPCR results show fold changes (mean ± SD, *n* = 2 or *n* = 4) relative to HSPA2+ RHE cultures. PCR primers are listed in Supplementary Table S1. **C** Immunoblots showing the levels of HSPA2, Casp1, and SLAMF7 proteins in cell lysates from HSPA2+ (CRISPR-CTR) and HSPA2- (CRISPR-2B, CRISPR-4) RHEs on days 3 and 18 of growth. Representative immunoblots are shown (*n* = 2). **D** Immunoblots showing the intracellular or extracellular (in concentrated conditioned medium) levels of SLAMF7 protein in control (HSPA2+) CRISPR-CTR and HSPA2- (CRISPR-2B; CRISPR-4) cells. Conditioned media were collected from cells cultivated in serum-free OptiMem medium. β-actin was used as a protein loading control. Graphs on the right side of immunoblots (in **C** and **D**) show results of the densitometry quantification of band intensity. Antibodies used in IHC and WB experiments are listed in Supplementary Table S2. In (**C**) and (**D**) 50–70 μg of protein sample per well was added. The Ponceau red-stained blot is shown as a protein loading control for a concentrated conditioned medium (18 µl was loaded per well). Representative immunoblots are shown (*n* = 2). Statistical significance of differences was calculated using a two-tailed *t*-test, *P ≤ 0.05.

Also, the mRNA levels of *Interferon Signaling* pathway-related genes (*IFITM3*, *IFIT2*, *IFIT3*, *EGR1*, *IFIH1*, *SLAMF7*, *TRANK1*, *IRF1*, *IRF7*, *IRF9*, *IFI16*) were increased in fully developed HSPA2- RHE (Fig. 7A, B). WB analysis of SLAMF7 (immune cells modulator) and Casp1 (inflammatory caspase) proteins revealed differences between HSPA2- and HSPA2+ RHEs. Casp1 levels were higher in less developed (day 3) HSPA2- RHE (Fig. 7C). SLAMF7 expression was detectable only in fully developed RHE and was higher in HSPA2- RHE (Fig. 7C). Furthermore, HSPA2- cells secreted higher levels of SLAMF7 when cultured in 2D (Fig. 7D). Altogether, our results indicate that the loss of HSPA2 is associated with increased release of proinflammatory mediators.

**Fig. 8:**
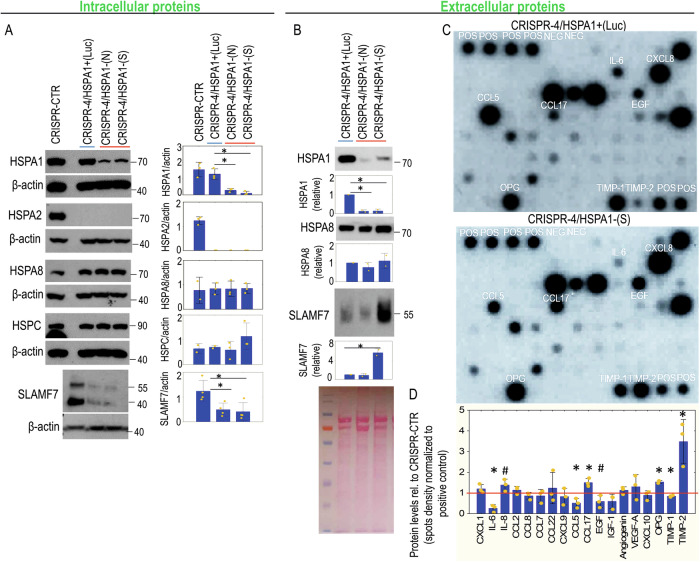
The effect of HSPA1 deficiency on the secretory profile of HSPA2-null HaCaT cells. **A** Representative immunoblots (*n* = 3) showing detection of HSPA1, HSPA2, HSPA8, HSPC, and SLAMF7 in control CRISPR-CTR (HSPA2+) cells, and in CRISPR-4 (HSPA2-) cells lentivirally transduced with non-targeting shRNA (CRISPR-4/HSPA1+(Luc)), or with HSPA1-targeting shRNA-N (CRISPR-4/HSPA1-(N)) or shRNA-S (CRISPR-4/HSPA1-(S)). β-actin was used as a protein loading control. Graphs on the right side of the immunoblots show the results of the densitometry quantification of band intensity. 35 or 70 μg (for SLAMF7 detection) of protein sample per well was added. **B** Extracellular (in conditioned media) levels of HSPA1, HSPA8, and SLAMF7 proteins in cells cultured under standard (2D) culture conditions. Graphs on the right side of the representative immunoblots (*n* = 2) show the results (mean ± SD) of the densitometry quantification of band intensity. Ponceau red-stained blot shows protein loading. 20 µl of concentrated medium was loaded per well. Conditioned media (in **B**, **C**) were collected from cells cultivated in serum-free OptiMem medium. **C** Extracellular levels of pro-inflammatory proteins (in conditioned media) detected by antibody array in cells growing under standard (2D) culture conditions. **D** The graph shows results (mean ± SD) of densitometric quantification of antibody microarray spots and represents the combined measurements for CRISPR-4/HSPA1-(N) and CRISPR-4/HSPA1-(S) cells (*n* = 2 for each variant) expressed relatively to CRISPR-4/HSPA1+ (Luc) cells after normalization to the internal positive control spots (POS; array spots of intensity ≤0.3 relative to POS were excluded from the analysis). The antibodies used in WB are listed in Supplementary Table S2. Statistical significance of differences between HSPA2-/HSPA1+ (CRISPR-4/HSPA1+ (Luc)) versus HSPA2-/HSPA1-deficient cells was calculated using a two-tailed *t*-test, *P ≤ 0.05. In (**D**) *P ≤ 0.05; #*P* ≤ 0.10.

Transcriptomic analysis revealed that HSPA2 loss affects TLR2/4 signaling pathways in keratinocytes (Fig. 3D), which express most TLRs [42]. HSPA2- cells secreted higher levels of eHSPA1 (Fig. 6G), a DAMP particle that activates TLR2/4 receptors to produce inflammatory mediators [43–45]. Therefore, we investigated whether reducing HSPA1 levels affects the secretory profile of HSPA2- cells. Stable *HSPA1A/B* knockdown in HSPA2- (CRISPR-4) cells (Fig. 8A) significantly reduced intracellular and extracellular HSPA1 levels (Fig. 8A, B) without compensatory increases of other chaperones (HSPA8, HSPC) (Fig. 8A, B). The antibody arrays showed that HSPA2-/HSPA1- cells grown in standard 2D culture had altered secretion compared to control HSPA2-/HSPA1+ cells, with reduced release of IL-6, CCL5, EGF, and TIMP-1, but increased IL-8, CCL17, OPG and TIMP-2 secretion (Fig. 8C, D). HSPA2-/HSPA1- cells also exhibited higher secretion of SLAMF7 (Fig. 8B), but reduced levels of intracellular SLAMF7 (Fig. 8A). These results suggest that the pro-inflammatory effects linked to HSPA2 loss are partially mediated by elevated eHSPA1 secretion.

## Discussion

In this study, we uncovered a novel complex homeostatic role of HSPA2 in epidermal keratinocytes. Using the RHE, a physiologically relevant human epidermis model in vitro, we found that the loss of HSPA2 led to underdevelopment of the granular layer and immunological alterations. Thus, inhibition of HSPA2 activity in keratinocytes can be considered an ‘intrinsic’ pathogenicity mechanism in the human epidermis [46]. The knowledge that changing a single molecule in keratinocytes can lead to aberrant differentiation and inflammation comes from studies on FLG or PPARα (Peroxisome Proliferators-Activated Receptors) (review in [46–50]. Currently, it is hard to conclude whether defects in terminal differentiation caused by HSPA2 loss led to immunological alterations, or conversely, the increased secretion of inflammatory factors by HSPA2- cells disrupts keratinocyte maturation.

Keratinocytes function as innate immune cells, serving as the first line of host defense and maintaining immune homeostasis, while also regulating adaptive immune responses in human skin. Due to production of a diverse range of immunomodulatory proteins, keratinocytes are often called ‘cytokinocytes’ [40, 51]. In this study, we demonstrated for the first time that HSPA2 can regulate the production and secretion of pro-inflammatory cytokines, chemokines, DAMP molecules, and expression of IFN-stimulated genes. We hypothesize that changes in HSPA2 levels may influence immune cell recruitment to the skin leading to dysfunctional immune responses [40, 52]. Therefore, our findings suggest that HSPA2 might play a role in the pathogenesis of inflammatory skin diseases.

In the skin, cytokines and chemokines control epidermal barrier homeostasis, facilitate wound healing, protect against viral and bacterial infection, and modulate immune responses [53–56]. The molecular mechanisms linking HSPA2 to pro-inflammatory mediators and IFN signaling remain unknown. Granular layer defects seem as unlikely cause, as HSPA2-null cells exhibited increased mRNA levels of IL-6 and chemokines regardless of cell culture type. Our findings suggest that the intracellular balance of HSPA2 and HSPA1 can regulate paracrine and autocrine signaling in keratinocytes. eHSPA1 mediates increased release of IL-6 and CCL5 in HSPA2- cells. This supports the immunomodulatory role of eHSPA1 [41, 57], which drives the production of inflammatory mediators that are likely to activate TLR signaling [45]. However, the pro-inflammatory phenotype of HSPA2- cells cannot be fully attributed to eHSPA1-dependent signaling, indicating the presence of additional factors within the HSPA2-regulated molecular network.

RHE transcriptomic profiling suggests that HSPA2 loss disrupts signaling pathways involving transcription factors critical to epidermal homeostasis and immune-related gene expression, including AP-1, ATF2, NF-κB, and STAT [58–61]. HSPA paralogs have been implicated in the modulation of pro-inflammatory gene expression by interacting with the activity of AP-1 and STAT transcription factors [62]. Intracellular HSPA1 as an anti-inflammatory factor can directly bind NF-κB subunits - key regulators of pro-inflammatory genes [63, 64] – to suppress NF-κB activity [65, 66]. Therefore, it seems reasonable to assume that HSPA2 could influence immunomodulatory signaling by modulating the activity of transcription factors. HIF-1α is the first confirmed HSPA2 client in keratinocytes [26]. However, functional analysis indicated that HIF-1 activity was unaffected by specific loss of HSPA2 in immortalized keratinocyte lines [26]. On the contrary, HIF-1 activity, regardless of oxygen availability, maintains a self-compensatory HSPA1/HSPA2/HSPA8-based system [26]. Thus, the functions of HIF-1 in regulating epidermal homeostasis [67–69] are sensitive to global HSPA inhibition. Recently, we have presented a conference report that EGFR can be a part of the HSPA2-regulated molecular network [70]. In this aspect, it is noteworthy that EGFR signaling can modulate chemokine expression in keratinocytes [71]. Therefore, we believe that HSPA2 plays pleiotropic roles and modulates distinct molecular pathways in keratinocytes. HSPA2 may control epidermal homeostasis by stabilizing the structure/activity of various client(s) (transcription factors, receptors, enzymes). Even subtle structural changes in multiple signaling proteins, co-occurring, can synergistically disrupt tissue homeostasis. Thus, further studies should comprehensively investigate the HSPA2-dependent molecular pathways.

We could not detect consistent transcriptomic changes in RHE with a high, although incomplete loss of HSPA2 expression (KD model) due to intra-sample variability. However, immunomodulatory signaling in the KD model might be affected by the activation of the IFN-mediated innate immune response induced by shRNA expression [72]. Recently, it was found that stimulation of the IFN-κ-mediated antiviral response in the immortalized keratinocyte N/TERT line (stably transduced with a hTERT-encoding retroviral vector [73]) following plasmid DNA transfection resulted in the permanent suppression of IFN-κ and IFN-stimulated gene expression due to the activation of epigenetic mechanism [24]. In our study, IFN-stimulated gene expression was sensitive to changes in HSPA2 levels in CRISPR/Cas9-modified cells. However, the findings from Sarkar et al., [24] suggest that avoiding viral vectors and recombinant DNA sequences (e.g., TERT or CDK4 genes) used for cell immortalization could be beneficial for research related to immunity.

Coordinated spatiotemporal regulation of keratinocyte differentiation is crucial for maintaining epidermal homeostasis. As observed in this and our previous study [18], reducing HSPA2 activity in basal keratinocytes below a certain threshold impaired the later stages of differentiation but did not affect the formation of basal and spinous layers in RHE. These observations suggest that the HSPA2-dependent chaperone platform can be active in certain keratinocyte population(s). This is possible as the expression patterns and function of HSPA2 and other HSPA paralogs do not fully overlap in the epidermis [18, 26]. Noteworthy, in keratinocytes HSPAs function has been linked to protection against thermal stress and UVB irradiation [21, 74].

The key finding of this study is that the loss of HSPA2 acts as an intrinsic pathogenicity mechanism leading to bidirectional functional changes in keratinocytes that impact both differentiation and immune signaling. Thus, HSPA2 could be implicated in cutaneous disease etiology and may represent a potential target or therapeutic agent in inflammatory skin diseases. Testing the efficacy of topically applied HSPA2 encapsulated in nanovesicles via a carrier (cream, patch) could offer a novel approach for treating inflammatory skin diseases. The potential of a similar strategy (a cream with plant-derived HSPAs) was reported to reduce psoriatic lesion development in mice [75].

**Fig. 9:**
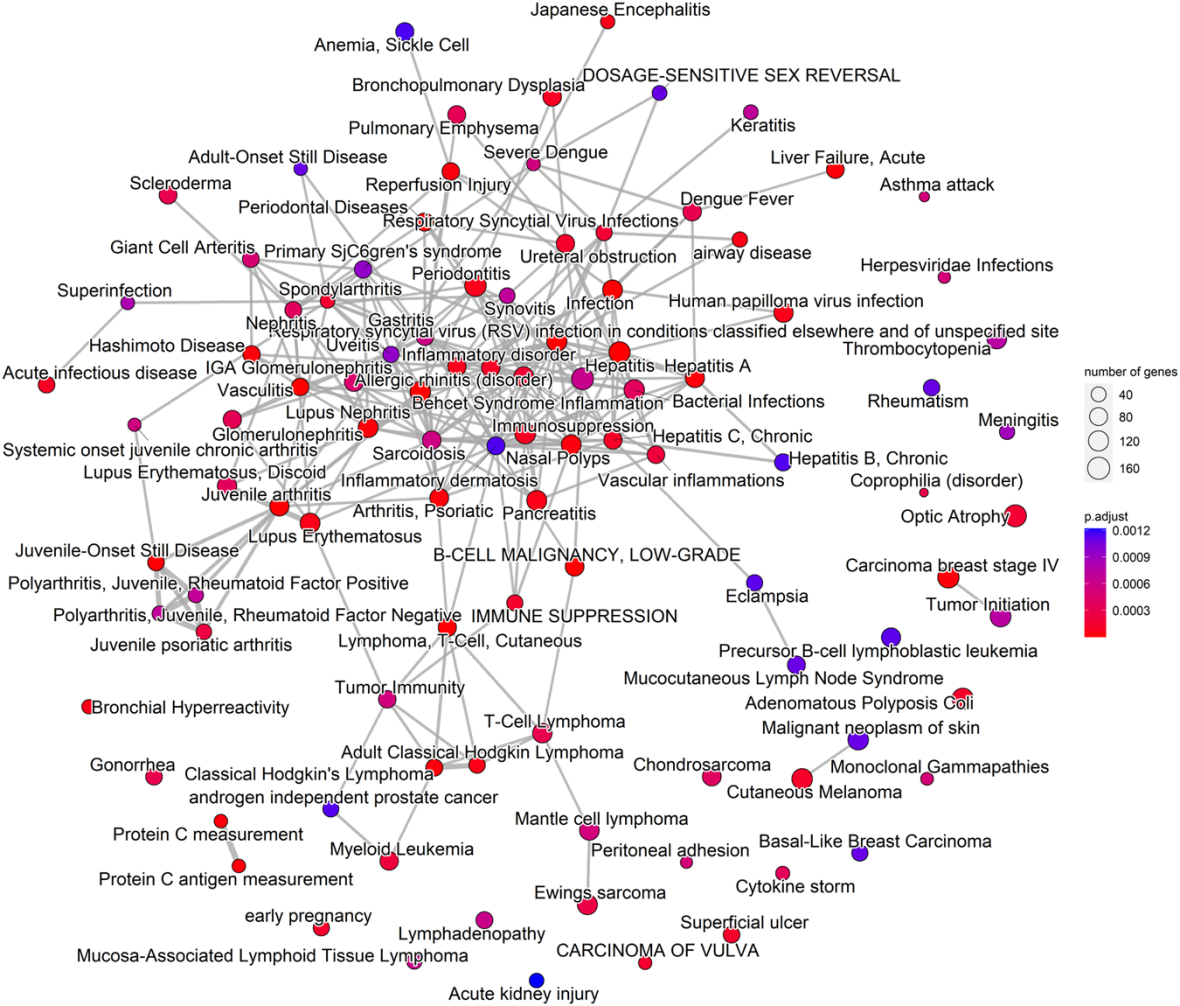
Gene-disease associations related to HSPA2 loss. Disease enrichment map obtained by performing GSEA for DisGeNET pathways (https://www.disgenet.org/) on all genes ranked according to the test statistic in supervised differential expression analysis for the KO model samples.

Using DisGeNET, a discovery platform containing one of the largest available collections of genes involved in human diseases, we observed that the gene expression profile associated with HSPA2 loss is also linked with a broad range of pathological states associated with inflammation (Fig. 9). Our results suggest that prolonged imbalance in the expression of HSPA paralogs in the epidermis may disrupt communication between epithelial and immune cells, potentially contributing to the development of inflammatory diseases. Therefore, further research on the role of HSPA2 in the pathogenesis of inflammatory skin diseases is warranted.

## Supplementary information

Supplementary Materials and Methods

Supplementary Results

Original data - Uncropped Western Blots

Original data - Raw qPCR Data

## Data Availability

The sequencing datasets involved in this work have been deposited in the ArrayExpress database under project accession numbers E-MTAB-13166 (KO in RHE), E-MTAB-13266 (KD in RHE), and E-MTAB-13251 (KO in 2D). The data that support other findings of this study are available from the corresponding author upon reasonable request.
