# Supplementary material for: HSPA2 influences the differentiation and production of immunomodulatory mediators in human immortalized epidermal keratinocyte lines
Source: Cell Death Dis. 2025 Apr 26;16(1):344. doi: 10.1038/s41419-025-07565-5 (PMC12033329; doi:10.1038/s41419-025-07565-5)
Supplement: Supplementary file 1 — Supplementary Materials and Methods [file 41419_2025_7565_MOESM1_ESM.pdf]

Supplementary Materials and Methods

HSPA2 influences the differentiation and production of immunomodulatory mediators in immortalized keratinocyte cell lines

Agnieszka Gogler[1]*, Agata Małgorzata Wilk[2,3]*, Damian Robert Sojka[1], Małgorzata Adamiec-Organiściok[1,3], Natalia Matysiak[4], Daria Kania[1], Klaudia Wiecha[1], Ewa Małusecka[1], Alexander Jorge Cortez[2], Dawid Zamojski[1,5,6], Michał Marczyk[5,7], Agnieszka Maria Mazurek[1], Sylwia Oziębło[1], Dorota Scieglinska#[1]

[1] Center for Translational Research and Molecular Biology of Cancer, Maria Sklodowska-Curie National Research Institute of Oncology Gliwice Branch, Wybrzeze Armii Krajowej 15, 44-102 Gliwice, Poland

[2] Department of Biostatistics and Bioinformatics, Maria Sklodowska-Curie National Research Institute of Oncology Gliwice Branch, Wybrzeze Armii Krajowej 15, 44-102 Gliwice, Poland

[3] Department of Systems Biology and Engineering, Silesian University of Technology, Akademicka 16, 44-100 Gliwice, Poland

[4] Department of Histology and Cell Pathology, Faculty of Medical Sciences in Zabrze, Medical University of Silesia in Katowice, Jordana 19, 41-808 Zabrze, Poland

[5] Department of Data Science and Engineering, Silesian University of Technology, Akademicka 16, 44-100 Gliwice, Poland

[6] Genetic Laboratory, Gyncentrum Sp. z o.o., 41-208 Sosnowiec, Poland

[7] Yale Cancer Center, Yale School of Medicine, New Haven, CT, USA

*These authors contributed equally to this work

Running title: Homeostatic role of HSPA2 in the human epidermis

# Corresponding author

Dorota Scieglinska, dorota.scieglinska@gliwice.nio.gov.pl; tel: (48) 32 27 89 679, ORCID 0000-0003-3489-8464

Reconstructed human epidermis model (RHE). To prepare fibroblast-seeded collagen gels, BJ1-hTERT fibroblasts (5×10^4 cells/insert) were resuspended in collagen I/collagen IV-supplemented DMEM/199 medium [2:1]. The membranes of Corning transwell® culture inserts (0.63 cm² area, 0.4 µm pores; Merck) were coated with a layer of fibroblast-collagen gel and submerged in a fibroblast growth medium for 5-7 days. RHE was generated as previously described [1].

HaCaT cells (20×10^4 cells/insert) were seeded on collagen-fibroblast-gel-coated inserts and maintained in standard DMEM/FBS medium in submerged culture for 3 days. Then, the cells were grown at the air-liquid interface (ALI) with the standard DMEM/FBS medium supplemented with 2 ng/ml TGFα (Merck) placed under the membrane. The medium was renewed every two days during the 18 days of ALI culture.

KerCT cells (22×10^4 cells/insert) were seeded on collagen-fibroblast-gel-coated inserts in a serum-free CnT-PRIME proliferation medium (CELLnTEC, Bern, Switzerland). The cultures were left submerged for 2 days. Then, cells were switched to a mixture of CnT-PRIME and DMEM high glucose medium [2:1], supplemented with 1,4 mM $CaCl_2$, 8 ng/ml cholera toxin, 40 µg/ml ascorbic acid, transferrin, insulin, epinephrine, rhEGF, bovine pituitary extract (BPE), and cultured submerged for 16-18 h. Next, the cultures were switched to the ALI culture for the next 18 days. The medium was renewed every two days during the 18 days of ALI culture.

RNA isolation and RT-PCR. Total RNA was extracted using the GeneJET RNA Purification Kit (Thermo Fisher Scientific, Waltham, MA, USA) following the manufacturer's protocol and purified from genomic DNA contamination as described previously [2]. cDNA was synthesized as described in [2]. Quantitative RT-qPCR was performed using a Bio-Rad C1000 Touch thermocycler connected to the CFX-96 head. Each reaction was executed in three technical repeats using PCR Master Mix SYBRGreen (A&A Biotechnology, Gdynia, Poland). Expression levels were normalized to *TMEM43* and *TBCB* unless otherwise stated. The nucleotide sequence of the primers is listed in Table S1.

Transcriptome Sequencing (RNA-Seq). Total RNA was isolated from fully developed RHE or a confluent monolayer (2D) culture using the GeneJET RNA Purification Kit (Thermo Fisher Scientific, Waltham, MA, USA) according to the manufacturer's protocol. Three biological replicates per genetic variant were sequenced and the replicates were cultured in three batches: RHE from the KD model, RHE from the KO model, and the KO model in 2D culture. RNA sequencing was outsourced to CeGaT GmbH (Tuebingen, Germany). cDNA libraries were prepared according to the standard Illumina protocol using the TruSeq Stranded mRNA library preparation kit sequenced by Illumina NovaSeq 6000 (run type: paired-end, read length: 2 × 100 bp).

RNA-Seq Data Analysis. Sequenced reads were demultiplexed using Illumina bcl2fastq v 2.20. The adapters were trimmed with Skewer v 0.2.2 [3] and the trimmed reads were aligned to the human hg19-cegat reference using STAR, v 2.7.3. [4] RNA-Seq data were analyzed using the R environment for statistical computing version 4.1.3 (R Foundation for Statistical Computing, Vienna, Austria). The quality of the RNA sample (RNA concentration, RIN), sequencing, and mapping quality were verified. After filtering low abundance genes (genes with total counts across all samples below 10), read counts were normalized using the median of ratios method implemented in DESeq2, version 1.34.0 [5], with the design accounting for the modification variant and biological replicate. Principal

component analysis (PCA) on unscaled, rlog-transformed expression levels was used to identify main sources of variability in the data, taking into account various subgroups of samples, i.e.: all samples cultured in RHE (six variants), samples of KD model cultured in RHE (three variants), all KO samples (RHE and 2D, six variants in total), samples of KO model grown in 2D culture (three variants), and KO model samples cultured in RHE (three variants). For the principal components that separated the samples into HSPA2+ and HSPA2- (PC3 in the KO model in RHE and PC5 in all KO model experiments), the top contributing genes were identifies with coefficients greater in magnitude than the threshold calculated according to the formula $WN^{-1/2}$, where N is the number of analyzed genes and W is a weight factor usually set to a value of minimum 3 (we assumed 3.5 for a more stringent criterion) [6, 7]. Over-representation analysis (ORA) was performed using the clusterProfiler package version 4.2.2 [8] to identify signaling pathways associated with the selected genes. Since many databases contain overlapping gene sets, the Reactome pathway database was used [9]. Differential expression analysis comparing the HSPA2+ and HSPA2- samples was executed in the subgroups described above. Reactome and disease-related pathways associated with the observed expression changes were analyzed using Gene Set Enrichment Analysis (GSEA), with genes ranked according to the Wald test statistic. In differential expression analysis and both types of pathway analysis (ORA, GSEA), P values < 0.05 were considered significant, after Benjamini-Hochberg correction for multiple testing.

Histomorphometric and ultrastructural analysis. Formalin-fixed and paraffin-embedded (FFPE) samples of RHE were prepared as described previously [1]. Quantitative histomorphometric analysis was performed on FFPE and HE-stained cross-sections (5 µm thick). To measure RHE thickness 7 - 9 random fields in each examined RHE specimen were selected and photographed with a 20× objective (a bright-field light microscope Axiophot equipped with an AxioCam 503 color camera and supported with AxioVision V4.6 software (Carl Zeiss AG, Germany). The RHE thickness in micrographs was analyzed using ImageJ software (RRID: SCR_003070).

The RHE ultrastructure was visualized by transmission electron microscopy (TEM). The samples were fixed in 4% glutaraldehyde in 0.1 M sodium cacodylate pH 7.4, postfixed with 1% osmium tetroxide in 0.1 M sodium cacodylate pH 7.4, and embedded in the EPON resin. 70-nm sections were imaged and photographed using the Tecnai G 2 Spirit Bio TWIN microscope (FEI Company, Hillsboro, OR, USA) equipped with the Rio 9 camera (Gatan Inc., Pleasanton, CA, USA).

Immunohistochemistry (IHC). IHC reactions were executed as described in [10]. The primary antibodies are listed in Table S2. IHC controls included the omission of the primary antibody. DAB staining was visualized and imaged using a bright-field light microscope.

Quantification of epidermal differentiation markers. Detection of color signal corresponding to DAB chromogen and quantification of areas occupied by DAB staining was based on the custom algorithm written in MATLAB® programming environment version 9.11.0.2022996 (R2021b) (The MathWorks Inc., Natick, MA, USA) as described in [11]. The pipeline included the following steps: (i) color normalization using Reinhard's method [12] to reduce the variability of pixel intensity values in different samples; (ii) color deconvolution using Ruifrok and Johnston method [13] to acquire color channels of the stains used; (iii) morphological operations to find the background area of the image; (iv) developed method for automatic image rotation based on boundary distribution of image intensity; and (v)

finding markers of human epidermal differentiation with k-means clustering. As a result, the staining proportion score (PS, %) was calculated as the percentage of the tissue area with a particular marker. For statistical analysis of the PS metric for each marker separately, the Kruskal-Wallis test was used. The post-hoc Nemenyi test was used to determine which groups were significantly different. In all tests, the statistical significance level was set to 0.05. Analysis was performed using R 4.4.0.

Protein extraction and Western blot (WB) analysis. Total cellular proteins were extracted using RIPA buffer (PBS, 1% NP-40, 0.1% SDS, 0.5% SDC, 50 mM NaF, 1 mM PMSF) supplemented with a protease inhibitor mixture (Roche Molecular Systems, Inc., Rotkreuz, Switzerland) as previously described [14]. Blots were generated using 25-35 µg of total protein if not indicated otherwise. For immunodetection, primary antibodies and HRP-conjugated secondary antibodies were used (Table S2). The chemiluminescent signal was developed using Clarity ECL Western Blot Substrate (Bio-Rad; Hercules, CA, USA), WesternBright Quantum, or Sirius HRP substrate (Advansta, San Jose, CA, USA), and imaged on X-ray film.

Proinflammatory stimulation and collection of conditioned media (CM). CM was collected from cells growing in 2D standard culture in a serum-free OptiMem medium, and untreated or treated with M5 cocktail (IL-17A, IL-1α, oncostatin M, TNFα, and IL-22; Bio Legend, San Diego, CA, USA) at a final concentration of 5 ng/ml for each cytokine. Next, CM samples were centrifuged to remove cell debris, then 30-fold concentrated using Vivaspin® concentrators with a molecular weight cutoff of 3 kDa (Sartorius, Paris, France). The samples of concentrated CM were stored (-70°C) and examined by WB.

An Antibody Array (Human Cytokine Array C3, RayBiotech, Peachtree Corners, GA, USA) was used to analyze inflammatory mediators in RHE and CM from RHE. Membranes were incubated with CM (1 ml) or RHE extracts (500 µg protein) overnight at 4°C. Clarity ECL Western Blot Substrate (Bio-Rad, Hercules, CA, USA) detected antibody-protein binding, and the chemiluminescent signal was imaged on X-ray film. Dot density was quantified using ImageJ software [15].

References

1. Gogler-Piglowska A, Klarzynska K, Sojka DR, Habryka A, Glowala-Kosinska M, Herok M, et al. Novel role for the testis-enriched HSPA2 protein in regulating epidermal keratinocyte differentiation. J Cell Physiol. 2018;233:2629-44.

2. Sojka DR, Gogler A, Kania D, Vydra N, Wiecha K, Adamiec-Organisciok M, et al. The human testis-enriched HSPA2 interacts with HIF-1alpha in epidermal keratinocytes, yet HIF-1alpha stability and HIF-1-dependent gene expression rely on the HSPA (HSP70) activity. Biochim Biophys Acta Mol Cell Res. 2024;1871:119735.

3. Jiang H, Lei R, Ding SW, Zhu S. Skewer: a fast and accurate adapter trimmer for next-generation sequencing paired-end reads. BMC Bioinformatics. 2014;15:182.

4. Dobin A, Davis CA, Schlesinger F, Drenkow J, Zaleski C, Jha S, et al. STAR: ultrafast universal RNA-seq aligner. Bioinformatics. 2013;29:15-21.

5. Love MI, Huber W, Anders S. Moderated estimation of fold change and dispersion for RNA-seq data with DESeq2. Genome Biol. 2014;15:550.

6. Wall ME, Dyck PA, Brettin TS. SVDMAN--singular value decomposition analysis of microarray data. Bioinformatics. 2001;17:566-8.

7. Simek K, Fujarewicz K, Swierniak A, Kimmel M, Jarzab B, Wiench M, et al. Using SVD and SVM methods for selection, classification, clustering and modeling of DNA microarray data. Eng Appl Artif Intel. 2004;17:417-27.

8. Wu TZ, Hu EQ, Xu SB, Chen MJ, Guo PF, Dai ZH, et al. clusterProfiler 4.0: A universal enrichment tool for interpreting omics data. Innovation (Camb). 2021;2:100141.

9. Milacic M, Beavers D, Conley P, Gong C, Gillespie M, Griss J, et al. The Reactome Pathway Knowledgebase 2024. Nucleic Acids Res. 2024;52:D672-D678.

10. Scieglinska D, Piglowski W, Chekan M, Mazurek A, Krawczyk Z. Differential expression of HSPA1 and HSPA2 proteins in human tissues; tissue microarray-based immunohistochemical study. Histochem Cell Biol. 2011;135:337-50.

11. Zamojski D, Gogler A, Scieglinska D, Marczyk M. EpidermaQuant: Unsupervised Detection and Quantification of Epidermal Differentiation Markers on H-DAB-Stained Images of Reconstructed Human Epidermis. Diagnostics. 2024;14:1904.

12. Khan AM, Rajpoot N, Treanor D, Magee D. A nonlinear mapping approach to stain normalization in digital histopathology images using image-specific color deconvolution. IEEE Trans Biomed Eng. 2014;61:1729-38.

13. Ruifrok AC, Johnston DA. Quantification of histochemical staining by color deconvolution. Anal Quant Cytol Histol. 2001;23:291-9.

14. Sojka DR, Gogler-Piglowska A, Vydra N, Cortez AJ, Filipczak PT, Krawczyk Z, et al. Functional redundancy of HSPA1, HSPA2 and other HSPA proteins in non-small cell lung carcinoma (NSCLC); an implication for NSCLC treatment. Sci Rep. 2019;9:14394.

15. Schneider CA, Rasband WS, Eliceiri KW. NIH Image to ImageJ: 25 years of image analysis. Nat Methods. 2012;9:671-5.

Table S1. Primers used in PCR

Gene	Annealing temperature (°C)	Nucleotide sequence	Product length (bp)
ABCA4	63	*F: 5′- TGAGCTGAAGAAGGCTGTGAGCTT -3′*<br>*R: 5′- TCGTCCCCTTCCGTGGGACT -3′*	135
ABCG1	61	*F: 5′- GGCACCGCCATGAATGC -3′*<br>*R: 5′- ATTCAGCAGGTCCGTCTCAG -3′*	120
CCL2	60.2	*F: 5′- AATCAATGCCCCAGTCACCT -3′*<br>*R: 5′- GGGTCAGCACAGATCTCCTT -3′*	153
CXCL1	61	*F: 5′- ACTGCGCCCAAACCGAAGTC -3′*<br>*R: 5′- TCTGGTCAGTTGGATTTGTCACTGT -3′*	127
EGR1	65	*F: 5′- GCACCTGACCGCAGAGTCTTTT -3′*<br>*R: 5′- ACTGACCAAGCTGAAGAGGGG -3′*	184
FOS	60	*F: 5′- TGTCAACGCGCAGGACTTCT -3′*<br>*R: 5′- AAAGGGTGAGGGGCTCTGGT -3′*	159
FOXO1	60.2	*F: 5′- AGGGTTAGTGAGCAGGTTACAC -3′*<br>*R: 5′- TGCTGCCAAGTCTGACGAAA -3′*	168
ICAM1	66.5	*F: 5′- AACCTGCCTTTCCCCAGAAG -3′*<br>*R: 5′- ACCGCTGAGTGTCATTGTGA -3′*	190
IFIT2	66.5	*F: 5′- AGGAAGGGTGGACACGGTTA -3′*<br>*R: 5′- TGCCTCAGAGGGTCAATGGC-3′*	178
IFIT3	54.5	*F: 5′- GGGCAGACTCTCAGATGCTC -3′*<br>*R: 5′- TCAAAACACACCTTCGCCCT -3′*	159
IFI16	67	*F: 5′- GAGCAAGCCAGCACTAGTCA -3′*<br>*R: 5′- CGGAACCGCAGGATGTTGTA -3′*	119
IL-6	65	*F: 5′- TACCCCCAGGAGAAGATTCC -3′*<br>*R: 5′- TTTTCTGCCAGTGCCTCTTT -3′*	175
IFITM3	54.5	*F: 5′- TAGGGACAGGAAGATGGTTGG -3′*<br>*R: 5′- GGATGACGATGAGCAGAATGG -3′*	122
IFIH1	64	*F: 5′- CTGCAAAAGAAGGAAATCGCA-3′*<br>*R: 5′- ACGCATCTATCATTCGAATTGTGT-3′*	109
IRF1	67	*F: 5′- AAGGAAAGTGGGGTCCTTCG -3′*<br>*R: 5′- ATGTGGCAAGATCCACACGA -3′*	124
IRF7	67.5	*F: 5′- CCATTCCTGGCACACACACA-3′*<br>*R: 5′- GGAAGCCCTTCTTGTCCCTC-3′*	188
IRF9	67	*F: 5′- TCCTCCAGAGCCAGACTACT -3′*<br>*R: 5′- CAATCCAGGCTTTGCACCTG -3′*	87
JUN	66	*F: 5′- GCCTCCAAGTGCCGAAAAAG -3′*<br>*R: 5′- GCAACTGCTGCGTTAGCATGA -3′*	190
KLK5	58	*F: 5′- AGTCAGAAAAGGTGCGAGGA-3′*<br>*R: 5′- TAATCTCCCCAGGACACGAG -3′*	155
KLK7	58	*F: 5′- CGCCGATGACCTATGAAGTCAAAT -3′*<br>*R: 5′- TGACTCTTCTCCAGCACTGAGGGT -3′*	155

KLK8	58	*F: 5′- CCAGAAGAAGTGTGAGGATG -3′*<br>*R: 5′- GGTATAGACGCCAGGTTTG -3′*	190
LCE3D	58	*F: 5′- CCCCATCTTGATGCATGAG -3′*<br>*R: 5′- TGTGACATCCTGGACATCAG -3′*	127
PTGS2	61	*F: 5′- ATATGTTCTCCTGCCTACTGGAA -3′*<br>*R: 5′- GCCCTTCACGTTATTGCAGATG -3′*	108
SOCS3	61	*F: 5′- GAAGATCCCCCTGGTGTTGA -3′*<br>*R: 5′- AAAGCGGGGCATCGTACTG -3′*	161
SLAMF7	67.5	*F: 5′- GGAAGATCCAGCAAATACGG -3′*<br>*R: 5′- GTTTTCTTTGGGCCGAGAAT -3′*	183
TBCB	60.2	*F: 5′-CCGAGAAGCGATACAGCCG-3′*<br>*R: 5′- CAGCCGTCATCTACAGGGTA-3′*	178
TMEM43	60.2	*F: 5′- CTTGTGGTGTCTCCCGACAG-3′*<br>*R: 5′- TTGGTACATCTCCACGTGCC-3′*	162
TRANK1	67.5	*F: 5′- GACAGCCACAGTGGAGTACC -3′*<br>*R: 5′- AGTGCCACTTCGCCCAATAA -3′*	109
WNT11	63	*F: 5′- CCGCCCGCGAGGTCATGAAG -3′*<br>*R: 5′- GACTCCCGGGTCCCTCTCTCC -3′*	118
WNT7B	61	*F: 5′- CTACTACAACCAAGCCGAGGG -3′*<br>*R: 5′- GGTCCTCTAGAACCTTCCTGC -3′*	167

Abbreviations: *ABCA4*, ATP Binding Cassette Subfamily A Member 4; *ABCG1*, ATP Binding Cassette Subfamily G Member 1; *CCL2*, C-C Motif Chemokine Ligand 2; *CXCL1*, CXC motif chemokine ligand 1; *EGR1*, Early Growth Response 1; *FOS*, Fos Proto-Oncogene, AP-1 Transcription Factor Subunit; *FOXO1*, Forkhead box protein O1; *ICAM1*, Intercellular Adhesion Molecule 1; *IFIT2*, Interferon Induced Protein With Tetratricopeptide Repeats 2; *IFIT3*, Interferon Induced Protein With Tetratricopeptide Repeats 3; *IFI16*, Interferon Alpha Inducible Protein 16; *IL-6*, Interleukin 6; *IFITM3*, Interferon Induced Transmembrane Protein 3; *IFIH1*, Interferon Induced With Helicase C Domain; *IRF1*, Interferon Regulatory Factor 1; *IRF7*, Interferon Regulatory Factor 7; *IRF9*, Interferon Regulatory Factor 9; *JUN*; Jun Proto-Oncogene, AP-1 Transcription Factor Subunit; *KLK5*, Kallikrein Related Peptidase 5; *KLK7*, Kallikrein Related Peptidase 7; *KLK8*, Kallikrein Related Peptidase 8, *LCE3D*, Late Cornified Envelope 3D; *PTGS2*, prostaglandin-endoperoxide synthase 2; *SOCS3*, Suppressor Of Cytokine Signaling 3; *SLAMF7*, SLAM Family Member 7; *TBCB*, Tubulin-folding Cofactor B; *TMEM43*, Transmembrane Protein 43; *TRANK1*, Tetratricopeptide Repeat And Ankyrin Repeat Containing 1; *WNT11*, Wnt Family Member 11; *WNT 7B*, Wnt Family Member 7B

Table S2. List of antibodies

Primary					
	**Host/Clonality**	**Clone**	**Catalog Number/ RRID**	**Source**	**Application (Dilution)**
**HSPA1**	Mo/M	C92F3A-5	ADI-SPA-810-F/AB_311860	Enzo, Life Sciences, Farmingdale, NY, USA	WB (1:6000)
**HSPA2**	Rb/M	EPR4596	Ab108416/AB_10862351	Abcam, Cambridge, UK	WB (1:6000) IHC/ICC (1:4000)
**HSPA8**	Mo/M	B-6	Sc-7298/AB_627761	Santa Cruz Biotechnology, Inc., TX, USA	WB (1:7500)
**HSP90/HSPC**	Mo/M	AC88	ADI-SPA-830-F/AB_11181197	Enzo, Life Sciences, Farmingdale, NY, USA	WB (1:2000)
**Filaggrin**	Mo/P		ab3137/AB_303542	Abcam, Cambridge, UK	IHC (1:50)
**Filaggrin**	Rb/P		905801/AB_2565053	BioLegend, San Diego, CA, USA	WB (1:500)
**Involucrin**	Rb/P		ab53112/ AB_880813	Abcam, Cambridge, UK	WB (1:1000) IHC (1:3000)
**Ki67**	Mo/M	MIB-1	M7240/AB_2142367	Dako-Agilent, Santa Clara, CA, USA	IHC (1:300)
**Keratin 10**	Rb/P		PRB-159P/AB_291580	BioLegend, San Diego, CA, USA	WB (1:1000) IHC (1:3000)
**p63**	Mo/M	DAK-p63	IR662/AB_2755007	Dako-Agilent, Santa Clara, CA, USA	RTU
**CCL2 (MCP-1)**	Mo/M	5-J	sc-32771/AB_626820	Santa Cruz Biotechnology, Inc., TX, USA	WB (1:500)
**CCL5 (RANTES)**	Mo/M	F-11	sc-514019	Santa Cruz Biotechnology, Inc., TX, USA	WB (1:500)
**Caspase 1**	Rb/M	EPR16883	ab179515/AB_2884954	Abcam, Cambridge, UK	WB (1:2000)
**IL-6**	Rb/P		ab6672/ AB_2127460	Abcam, Cambridge, UK	WB (1:1500)
**SLAMF7**	Rb/M	E5C4M	98611/AB_2800306	Cell Signaling Technology, Inc., Danvers, MA, USA	WB (1:1000-1:2000)

**β-actin (HRP-conjugated)**	Mo/M	AC15	A3854/AB_262011	Merck Millipore KGaA, Darmstadt, Germany	WB (1:25000)
**Secondary**					
**Anti-Mo IgG (HRP-conjugated)**	Go		AP124P/AB_90456	Millipore, Billerica, MA, USA	WB (1:5000)
**Anti-Rb IgG (HRP-conjugated)**	Go		AP132P/AB_90264	Millipore, Billerica, MA, USA	WB (1:2000)

Abbreviations: RRID, Research Resource Identifier; WB, Western blot; IHC, immunohistochemistry; ICC, immunocytochemistry; M, monoclonal; P, polyclonal; Go, goat; Mo, mouse; Rb, rabbit; HRP, horseradish peroxidase
