# Supplementary material for: HSPA2 influences the differentiation and production of immunomodulatory mediators in human immortalized epidermal keratinocyte lines
Source: Cell Death Dis. 2025 Apr 26;16(1):344. doi: 10.1038/s41419-025-07565-5 (PMC12033329; doi:10.1038/s41419-025-07565-5)
Supplement: Supplementary file 2 — Supplementary Results [file 41419_2025_7565_MOESM2_ESM.pdf]

Supplementary Results

HSPA2 influences the differentiation and production of immunomodulatory mediators in immortalized keratinocyte cell lines

Agnieszka Gogler[1]*, Agata Małgorzata Wilk[2,3]*, Damian Robert Sojka[1], Małgorzata Adamiec-Organiściok[1,3], Natalia Matysiak[4], Daria Kania[1], Klaudia Wiecha[1], Ewa Małusecka[1], Alexander Jorge Cortez[2], Dawid Zamojski[1,5,6], Michał Marczyk[5,7], Agnieszka Maria Mazurek[1], Sylwia Oziębło[1], Dorota Scieglinska#[1]

[1] Center for Translational Research and Molecular Biology of Cancer, Maria Sklodowska-Curie National Research Institute of Oncology Gliwice Branch, Wybrzeze Armii Krajowej 15, 44-102 Gliwice, Poland

[2] Department of Biostatistics and Bioinformatics, Maria Sklodowska-Curie National Research Institute of Oncology Gliwice Branch, Wybrzeze Armii Krajowej 15, 44-102 Gliwice, Poland

[3] Department of Systems Biology and Engineering, Silesian University of Technology, Akademicka 16, 44-100 Gliwice, Poland

[4] Department of Histology and Cell Pathology, Faculty of Medical Sciences in Zabrze, Medical University of Silesia in Katowice, Jordana 19, 41-808 Zabrze, Poland

[5] Department of Data Science and Engineering, Silesian University of Technology, Akademicka 16, 44-100 Gliwice, Poland

[6] Genetic Laboratory, Gyncentrum Sp. z o.o., 41-208 Sosnowiec, Poland

[7] Yale Cancer Center, Yale School of Medicine, New Haven, CT, USA

*These authors contributed equally to this work

Running title: Homeostatic role of HSPA2 in the human epidermis

Corresponding author

Dorota Scieglinska, dorota.scieglinska@gliwice.nio.gov.pl; tel: (48) 32 27 89 679, ORCID 0000-0003-3489-8464

Figure S1.

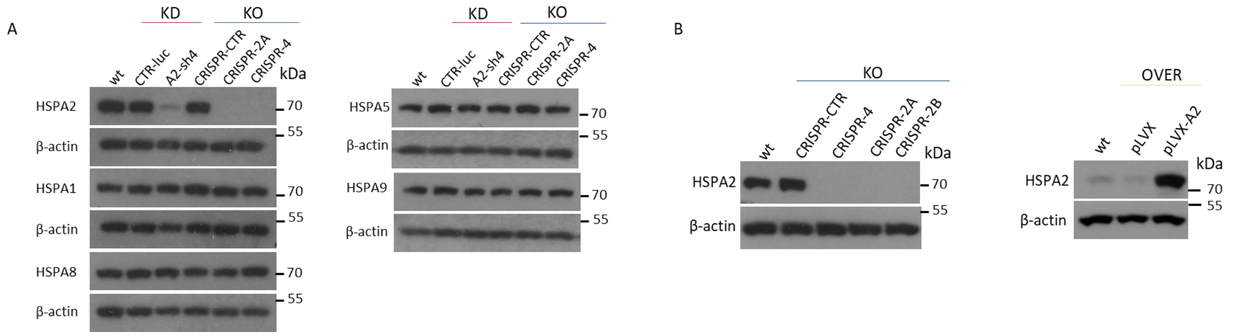

Figure S1. (A) Expression of the HSPA paralogs in HSPA2+ and HSPA2- variants of HaCaT cells. In the knockdown (KD) model, the HSPA2- variant is represented by A2-sh4 cells, while the control HSPA2+ is by CTR-luc cells. In the knockout (KO) model, HSPA2- variants are represented by CRISPR-2A and CRISPR-4 cells, while HSPA2+ by the control CRISPR-CTR cells. "wt" indicates wild-type cells. Representative immunoblots are shown (n = 2). **(B)** Representative immunoblots showing levels of HSPA2 in wt cells, and in cells of KO model and HSPA2-overexpression (OVER) model (HSPA2+, pLVX; HSPA2-OVER, pLVX-A2). β-actin was used as a protein loading control, blots were generated using 25 - 35 μg of total protein per well.

Figure S2.

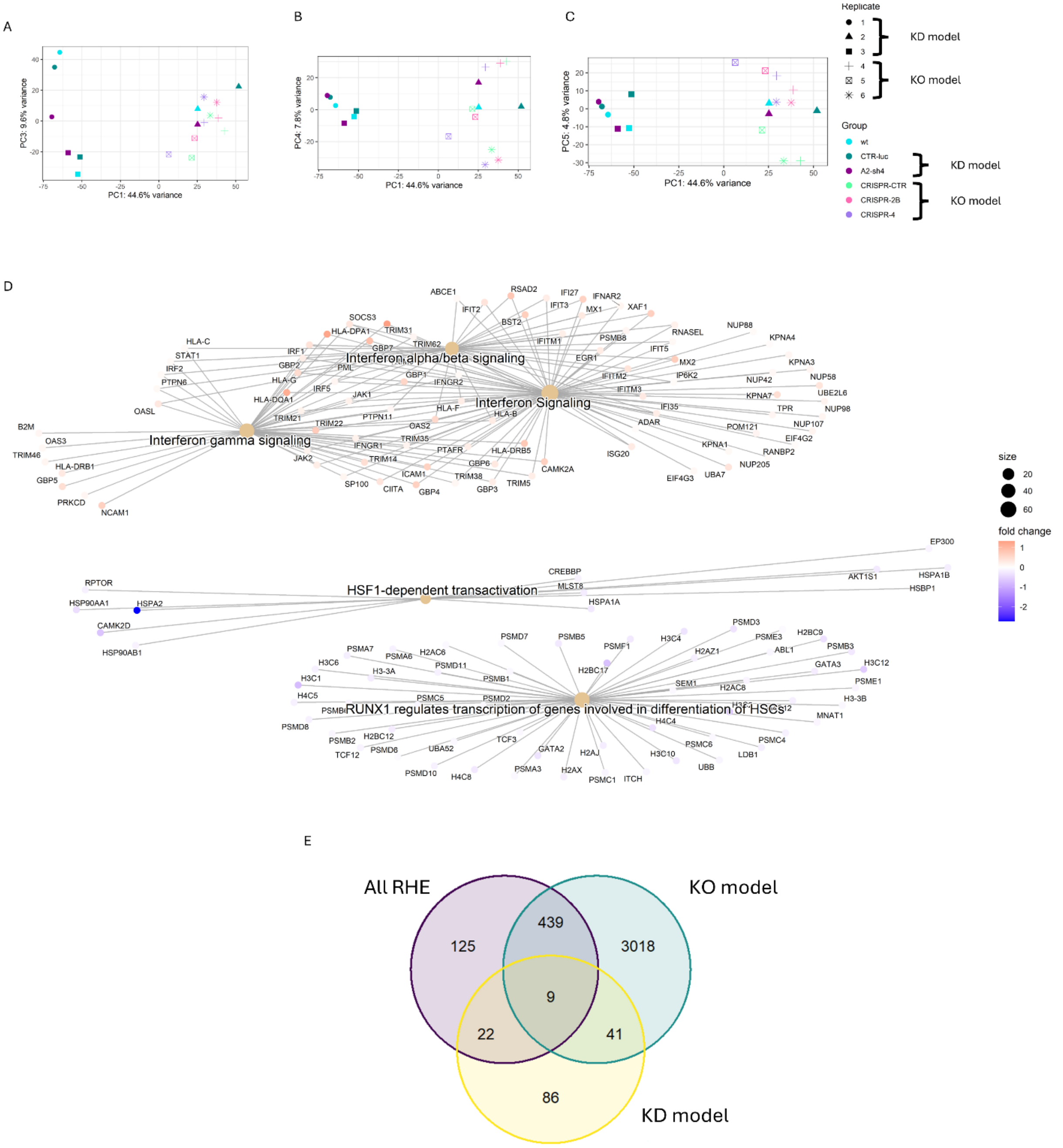

Figure S2. Transcriptomic comparison of the reconstructed human epidermis (RHE) cultures formed by HSPA2+ and HSPA2- HaCaT cells. Datasets from KD (knockdown) and KO (knockout) cell models and from wild-type cells were analyzed together. **(A-C)** Principal Component Analysis (PCA) of unscaled, rlog-transformed gene expression levels in 18-day RHE cultures. In the KD model, HSPA2- RHE is represented by the A2-sh4 cells (group; purple), while HSPA2+ by the CTR-luc cell variant (dark green); in the KO model, HSPA2- RHE is represented by CRISPR-2B (pink) and CRISPR-4 (violet) cells, while HSPA2+ by CRISPR-CTR (neon green) cells. RHE formed by wild-type

cells (wt, blue) was considered an additional variant of HSPA2+ cells. **(D)** The annotated gene-concept network shows the top 5 enriched pathways identified in the GSEA analysis. Fold changes (log2) in HSPA2- versus HSPA2+ RHEs in the expression of core enrichment-contributing genes are indicated with colors. Upregulated genes are shown in red, while downregulated ones in blue. The top enriched pathways in HSPA2- RHEs were linked to interferon signaling, cell differentiation, and HSF-1-dependent transactivation. In the latter case, a decrease in the expression of the major stress-inducible *HSPA1A/B*, *HSP90AA1/AB1*, and *HSPB1* genes was observed. **(E)** The Venn diagram depicts differentially expressed genes (DEGs) in HSPA2- versus HSPA2+ for all RHE samples (wt, KD, and KO models) (purple), and compared with DEGs obtained by analyzing the KD (yellow) and KO (green) models, independently. Comparing gene expression levels, inconsistent differences between the KD and KO models for the majority of DEGs were found. Only 9 DEGs were common for all three comparisons, while the vast majority of DEGs were found in only one of the models.

Figure S3.

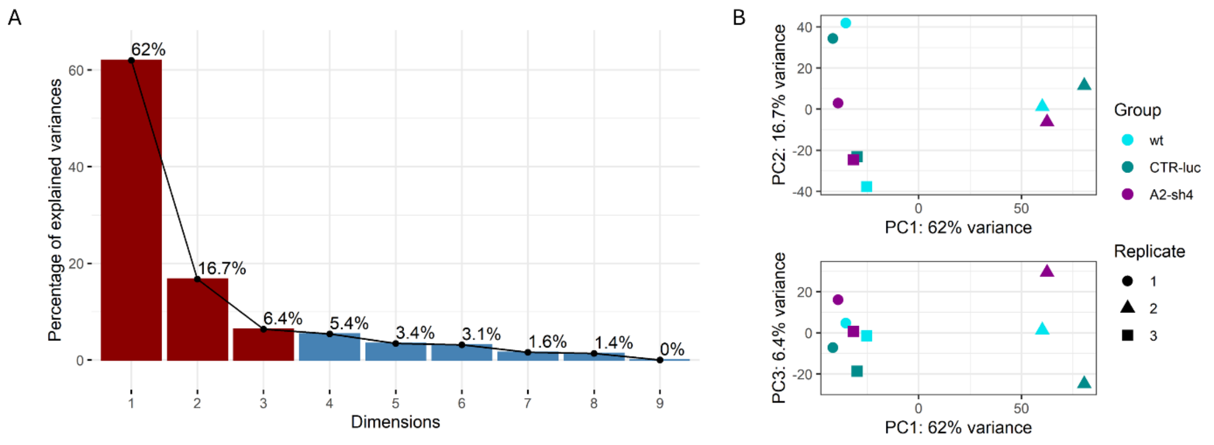

Figure S3. Transcriptomic comparison of HSPA2- versus HSPA2+ cells of the knockdown (KD) model grown as the reconstructed human epidermis (RHE). **(A)** The percentage of variance explained by consecutive principal components (PC) in Principal Component Analysis (PCA) on rlog-transformed, unscaled expression levels of all genes. Dark red indicates PCs responsible for 80% of the total variance. **(B)** Two-dimensional scatter plot of scores for the first three PCs from the PCA. Colors represent cell modification variants, and shapes represent replicates. HSPA2- RHE is represented by A2-sh4 cells (purple); HSPA2+ by CTR-luc cells (dark green), and additionally by wild-type (wt) cells. The variability observed in the data with PCA analysis was primarily related to biological replications. HSPA2- (A2-sh4) RHEs show no separation from HSPA2+ (wt, CTR-luc) RHEs.

Figure S4

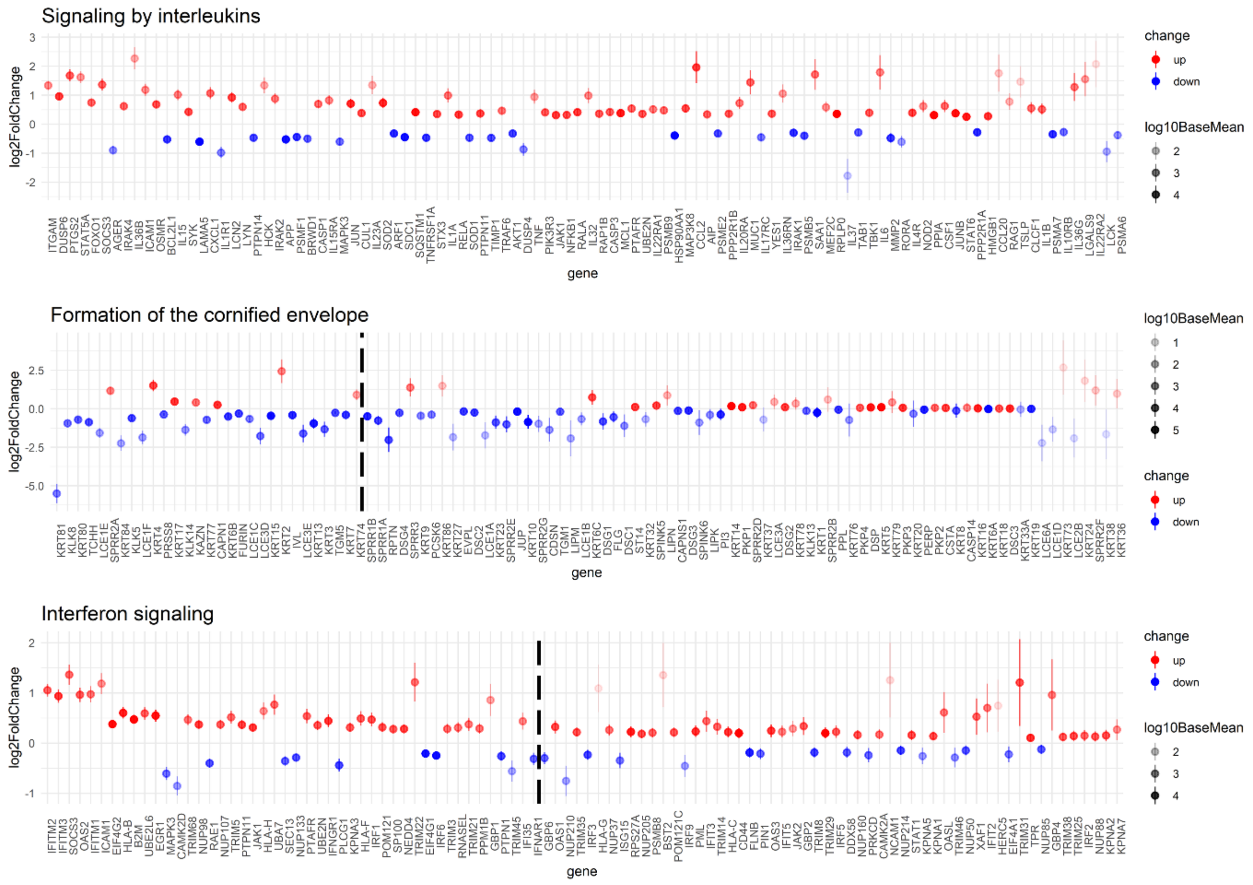

Figure S4. Gene expression in the top three enriched pathways in HSPA2- versus HSPA2+ reconstructed human epidermis (RHE) (the knockout (KO) model). The top 100 genes are shown according to the adjusted p-value. The y-axis shows log2FoldChange between HSPA2- and HSPA2+ cells. Upregulated genes are shown in red and downregulated genes in blue. Marker opacity represents the average expression level. The black vertical line shows the significance threshold (Padj = 0.05). In the case of the *Signaling by interleukins* pathway, more than 100 genes were significant.

Figure S5.

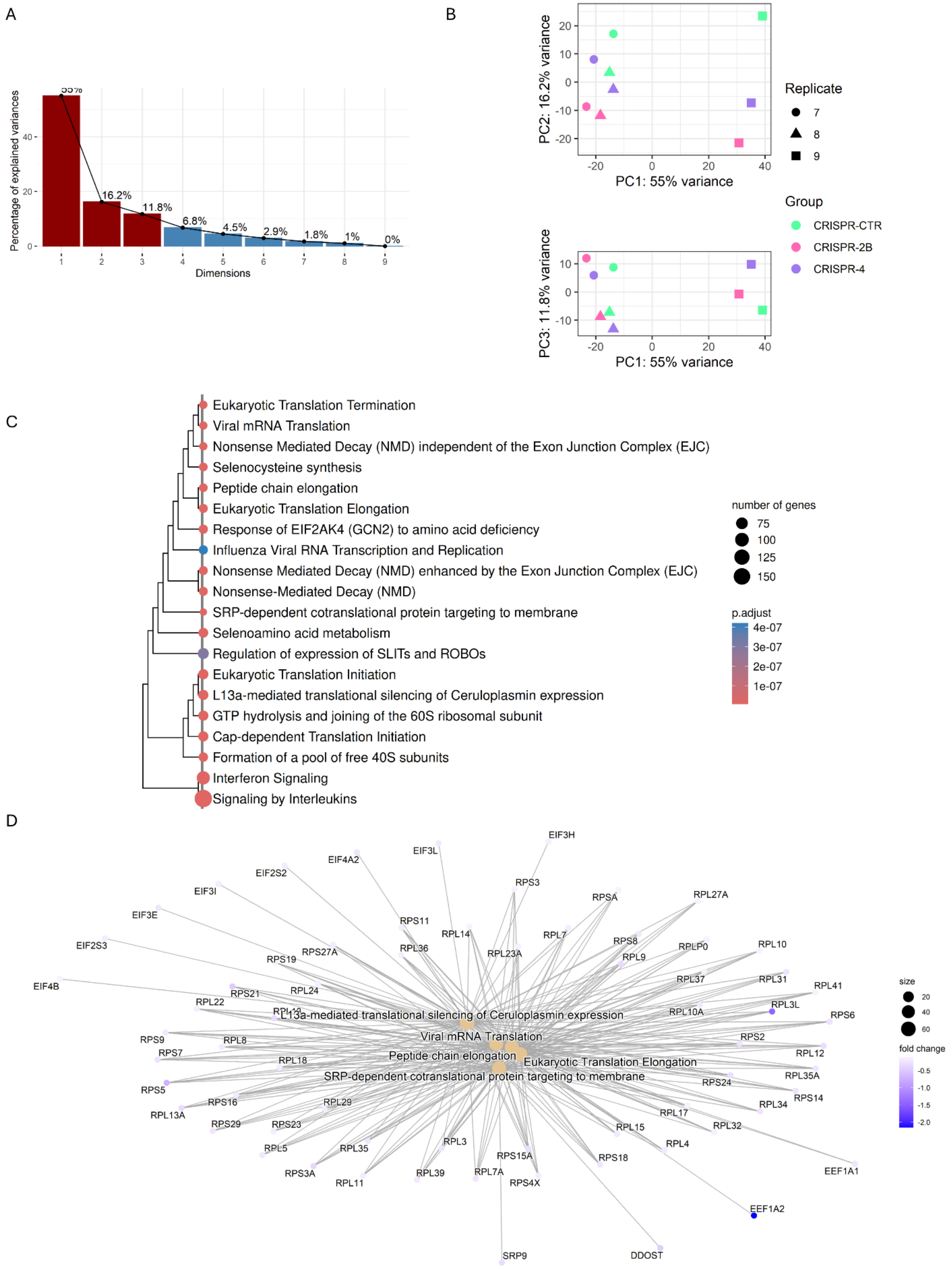
A
B
C
D
Percentage of explained variances
Dimensions
55%
16.2%
11.8%
6.8%
4.5%
2.9%
1.8%
1%
0%
PC2: 16.2% variance
PC1: 55% variance
PC3: 11.8% variance
Replicate
7
8
9
Group
CRISPR-CTR
CRISPR-2B
CRISPR-4
Eukaryotic Translation Termination
Viral mRNA Translation
Nonsense Mediated Decay (NMD) independent of the Exon Junction Complex (EJC)
Selenocysteine synthesis
Peptide chain elongation
Eukaryotic Translation Elongation
Response of EIF2AK4 (GCN2) to amino acid deficiency
Influenza Viral RNA Transcription and Replication
Nonsense Mediated Decay (NMD) enhanced by the Exon Junction Complex (EJC)
Nonsense-Mediated Decay (NMD)
SRP-dependent cotranslational protein targeting to membrane
Selenoamino acid metabolism
Regulation of expression of SLITs and ROBOs
Eukaryotic Translation Initiation
L13a-mediated translational silencing of Ceruloplasmin expression
GTP hydrolysis and joining of the 60S ribosomal subunit
Cap-dependent Translation Initiation
Formation of a pool of free 40S subunits
Interferon Signaling
Signaling by Interleukins
number of genes
75
100
125
150
p.adjust
4e-07
3e-07
2e-07
1e-07
L13a-mediated translational silencing of Ceruloplasmin expression
Viral mRNA Translation
Peptide chain elongation
Eukaryotic Translation Elongation
SRP-dependent cotranslational protein targeting to membrane
size
20
40
60
fold change
-0.5
-1.0
-1.5
-2.0
EIF3H
EIF3L
EIF4A2
EIF2S2
EIF3I
EIF3E
EIF2S3
EIF4B
RPS3
RPSA
RPL27A
RPS11
RPL14
RPL7
RPS8
RPLP0
RPL10
RPL36
RPL23A
RPL9
RPS19
RPS27A
RPL37
RPL31
RPL41
RPS21
RPL24
RPL22
RPL10A
RPL3L
RPS6
RPS9
RPL8
RPS2
RPS7
RPL18
RPL12
RPL35A
RPS24
RPS5
RPS16
RPL29
RPS14
RPL34
RPL13A
RPS29
RPS23
RPL17
RPL32
RPL15
RPL4
RPL5
RPL35
RPL3
RPS15A
RPS18
EEF1A1
RPS3A
RPL11
RPL39
RPL7A
RPS4X
EEF1A2
SRP9
DDOST

Figure S5. Transcriptomic comparison of gene expression in HSPA2- versus HSPA2+ cells cultured under standard 2D conditions (the knockout (KO) model). **(A)** The percentage of variance explained by consecutive principal components (PC) in principal component analysis (PCA) performed on rlog-transformed, unscaled expression levels of all genes. Dark red indicates components sufficient to explain 80% of the total variance. **(B)** Two-dimensional scatter plot of scores for the first three PC from the PCA. Colors represent cell modification variants, and shapes represent replicates. HSPA2- RHE is represented by CRISPR-2B (pink) and CRISPR-4 (violet) cells; HSPA2+ by CRISPR-CTR (neon green). The variability observed in the data after PCA was primarily related to biological replications. **(C)** Supervised (GSEA) analysis comparing gene expression in HSPA2- versus HSPA2+ cells. Treeplot shows hierarchical clustering of the top 20 enriched terms. **(D)** The annotated gene concept-network shows the top five enriched pathways. Enriched pathways in HSPA2- cells were related to the translation process (and were downregulated). Fold changes (log2) are illustrated with colors, upregulated genes are shown in red, while downregulated in blue.

Figure S6.

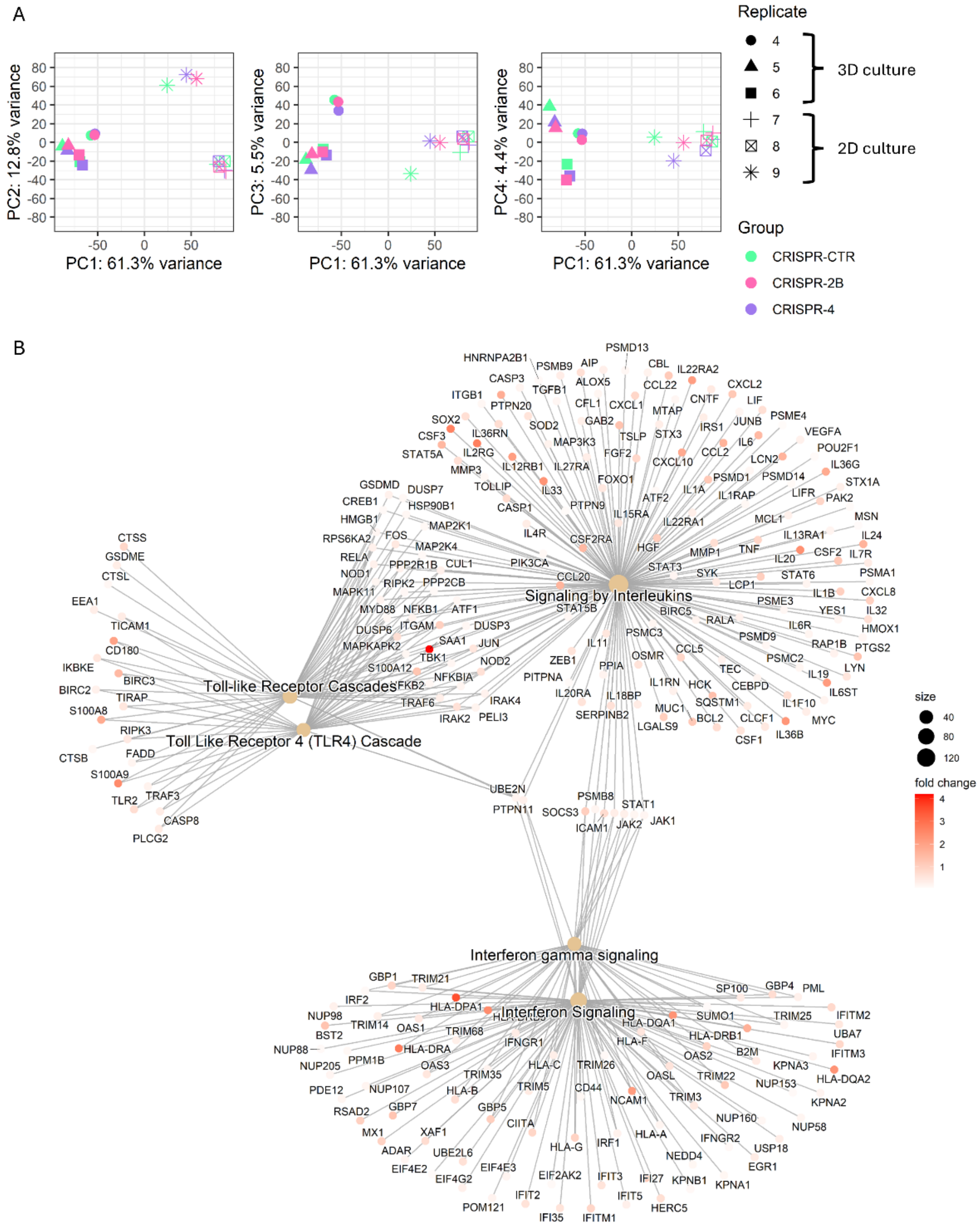

Figure S6. Transcriptomic comparison of HSPA2- versus HSPA2+ cells cultured in standard 2D (replicates 7-9) or RHE (replicates 4-6) cultures (the knockout (KO) model). **(A)** Principal Component Analysis (PCA) of unscaled, rlog-transformed gene expression levels. Two-dimensional scatter plots show scores for the first four principal components (PC). Colors represent cell modification variants, and shapes represent replicates. HSPA2- RHE is represented by CRISPR-2B (pink) and CRISPR-4 (violet) cells; HSPA2+ by CRISPR-CTR (neon green). **(B)** Annotated gene-concept network shows the top 5 enriched pathways in the GSEA. Fold changes (log2) in HSPA2- versus HSPA2+ cells for core enrichment-contributing genes are indicated with colors. Upregulated genes are shown in red, while downregulated in blue.

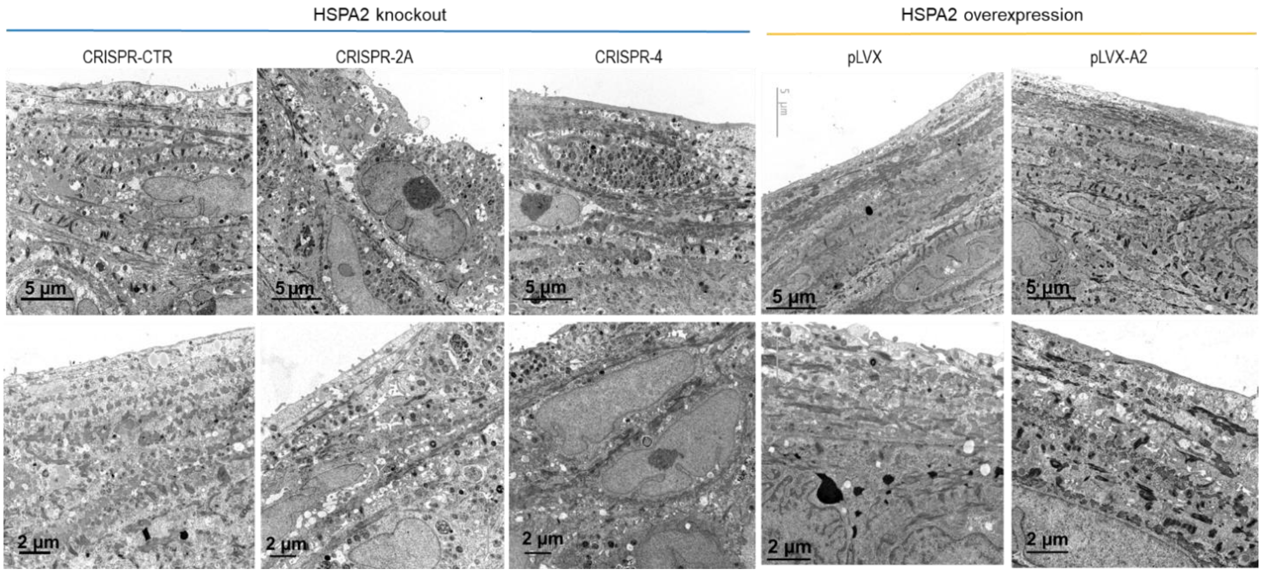

Figure S7. Representative transmission electron microscopy microphotographs of 18-day RHE cultures formed by control HSPA2+ (CRISPR-CTR, pLVX) and HSPA2- (CRISPR2A, CRISPR-4) HaCaT cells. The scale bar represents magnification. The imaging was performed for two independent RHE cultures in each group.
