# Supplementary material for: HSPA2 influences the differentiation and production of immunomodulatory mediators in human immortalized epidermal keratinocyte lines
Source: Cell Death Dis. 2025 Apr 26;16(1):344. doi: 10.1038/s41419-025-07565-5 (PMC12033329; doi:10.1038/s41419-025-07565-5)

Original Data – Uncropped Western Blot Images

HSPA2 influences the differentiation and production of immunomodulatory mediators in human immortalized epidermal keratinocyte lines

Agnieszka Gogler[1]*, Agata Małgorzata Wilk[2,3]*, Damian Robert Sojka[1], Małgorzata Adamiec-Organiściok[1,3], Natalia Matysiak[4], Daria Kania[1], Klaudia Wiecha[1], Ewa Małusecka[1], Alexander Jorge Cortez[2], Dawid Zamojski[1,5,6], Michał Marczyk[5,7], Agnieszka Maria Mazurek[1], Sylwia Oziębło[1], Dorota Scieglinska#[1]

[1] Center for Translational Research and Molecular Biology of Cancer, Maria Sklodowska-Curie National Research Institute of Oncology Gliwice Branch, Wybrzeze Armii Krajowej 15, 44-102 Gliwice, Poland

[2] Department of Biostatistics and Bioinformatics, Maria Sklodowska-Curie National Research Institute of Oncology, Gliwice Branch, Wybrzeze Armii Krajowej 15, 44-102 Gliwice, Poland

[3] Department of Systems Biology and Engineering, Silesian University of Technology, Akademicka 16, 44-100 Gliwice, Poland

[4] Department of Histology and Cell Pathology, Faculty of Medical Sciences in Zabrze, Medical University of Silesia in Katowice, Jordana 19, 41-808 Zabrze, Poland

[5] Department of Data Science and Engineering, Silesian University of Technology, Akademicka 16, 44-100 Gliwice

[6] Genetic Laboratory, Gyncentrum Sp. z o.o., 41-208 Sosnowiec, Poland

[7] Yale Cancer Center, Yale School of Medicine, New Haven, CT, USA

*These authors contributed equally to this work

Running title: Homeostatic role of HSPA2 in the human epidermis

Corresponding author

Dorota Scieglinska, dorota.scieglinska@gliwice.nio.gov.pl; tel: (48) 32 27 89 679, ORCID 0000-0003-3489-8464

Fig 4G

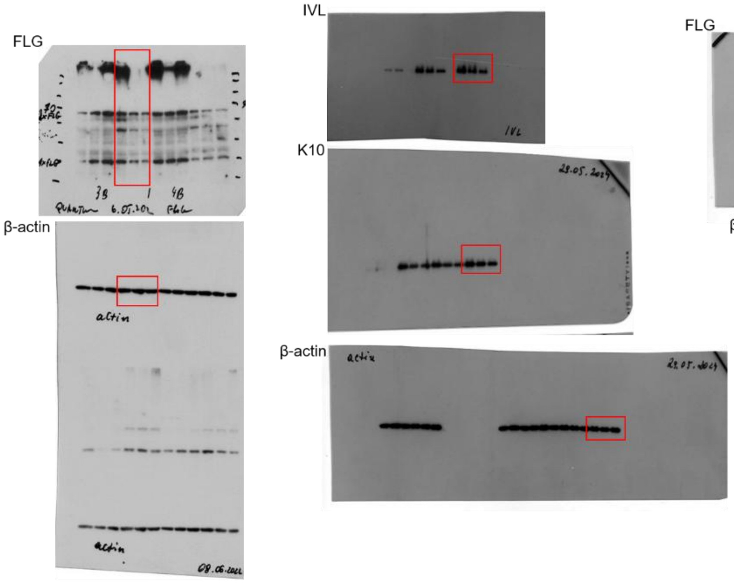

Fig 4M

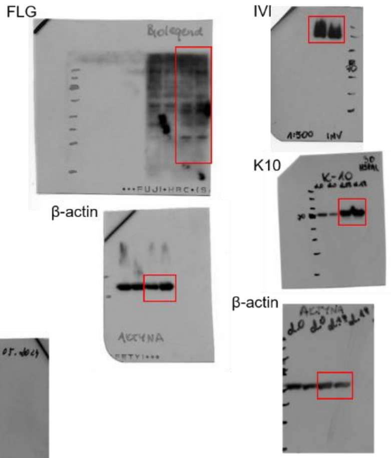

Fig 5A

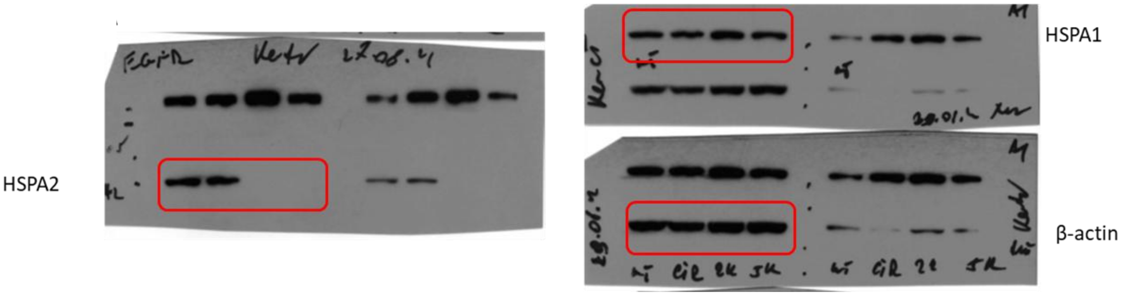

Fig 6G

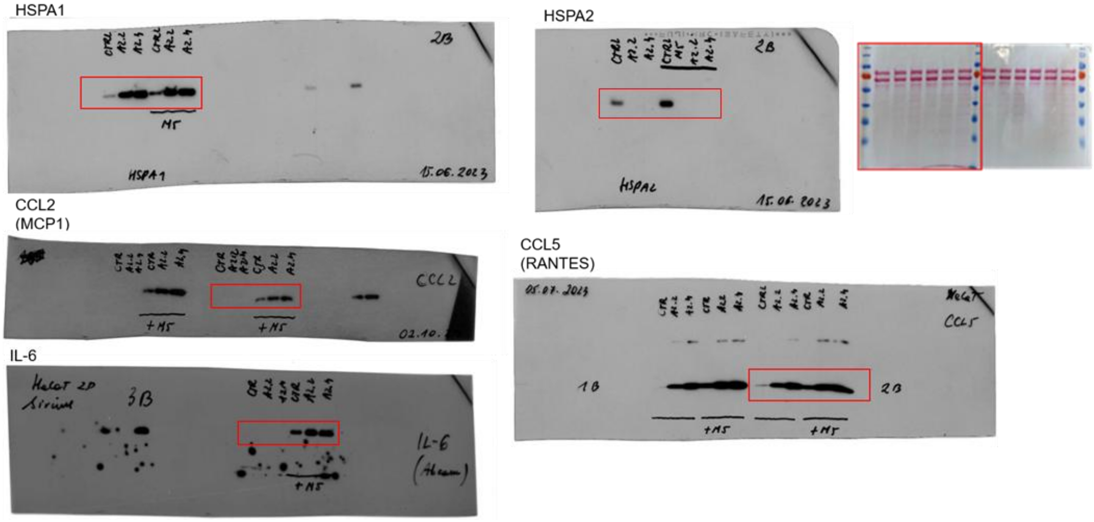

Fig 7C

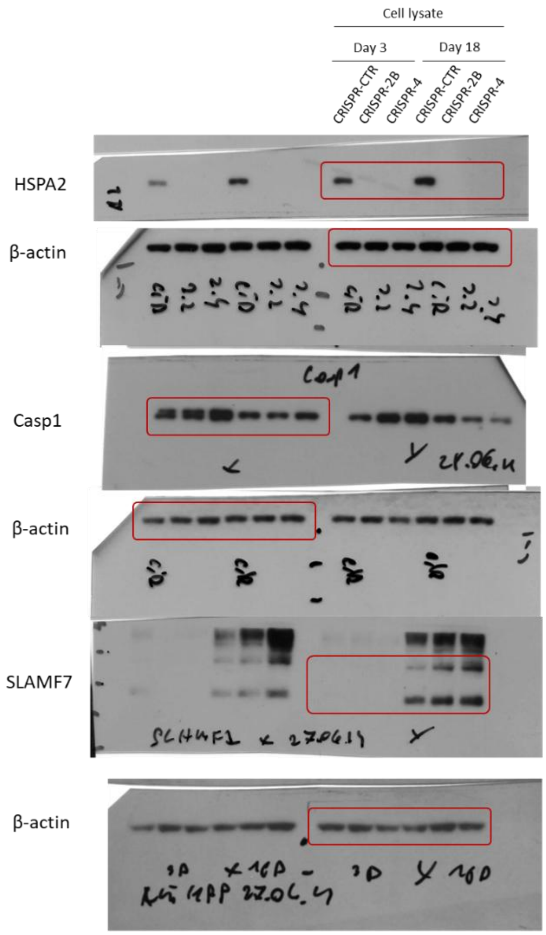
Cell lysate
Day 3
Day 18
CRISPR-CTR
CRISPR-2B
CRISPR-4
CRISPR-CTR
CRISPR-2B
CRISPR-4
HSPA2
β-actin
Casp1
β-actin
SLAMF7
β-actin

Fig 7D

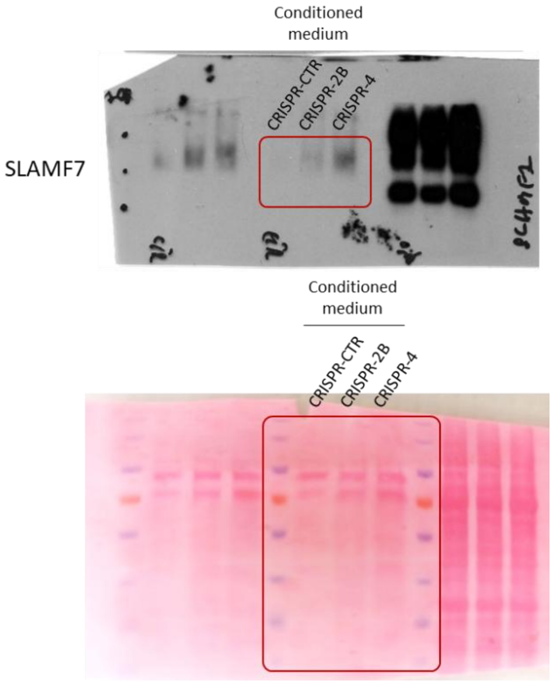
Conditioned medium
CRISPR-CTR
CRISPR-2B
CRISPR-4
SLAMF7
Conditioned medium
CRISPR-CTR
CRISPR-2B
CRISPR-4

Fig 8A

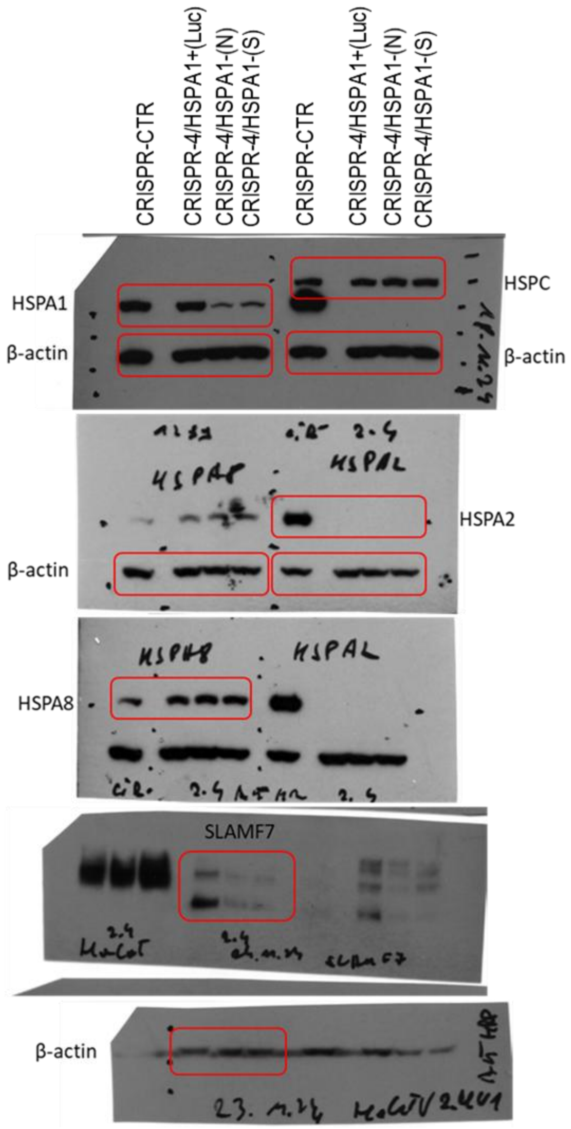
CRISPR-CTR
CRISPR-4/HSPA1+(Luc)
CRISPR-4/HSPA1-(N)
CRISPR-4/HSPA1-(S)
CRISPR-CTR
CRISPR-4/HSPA1+(Luc)
CRISPR-4/HSPA1-(N)
CRISPR-4/HSPA1-(S)
HSPA1
β-actin
HSPC
β-actin
HSPA2
β-actin
HSPA8
SLAMF7
β-actin

Fig 8B

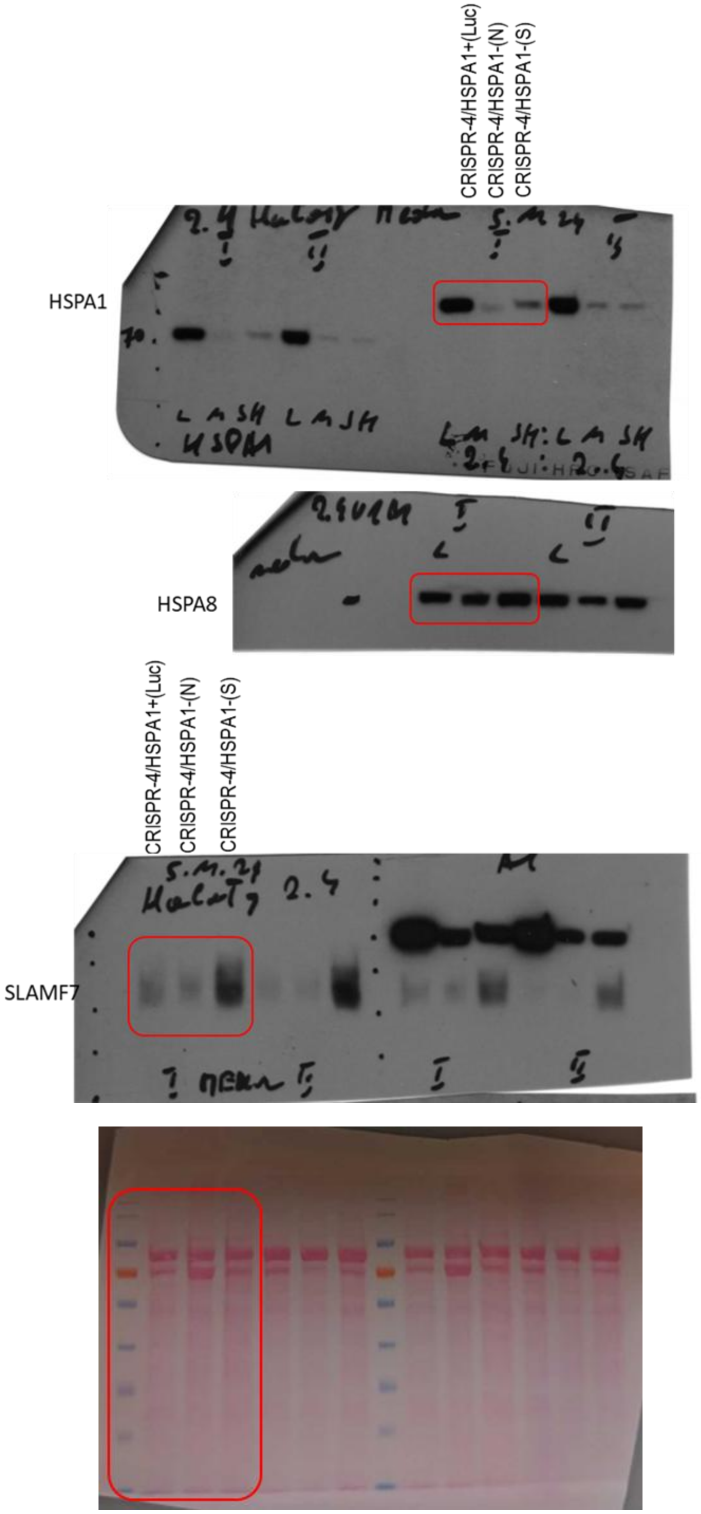
CRISPR-4/HSPA1+(Luc)
CRISPR-4/HSPA1-(N)
CRISPR-4/HSPA1-(S)
HSPA1
HSPA8
CRISPR-4/HSPA1+(Luc)
CRISPR-4/HSPA1-(N)
CRISPR-4/HSPA1-(S)
SLAMF7

Fig S1A

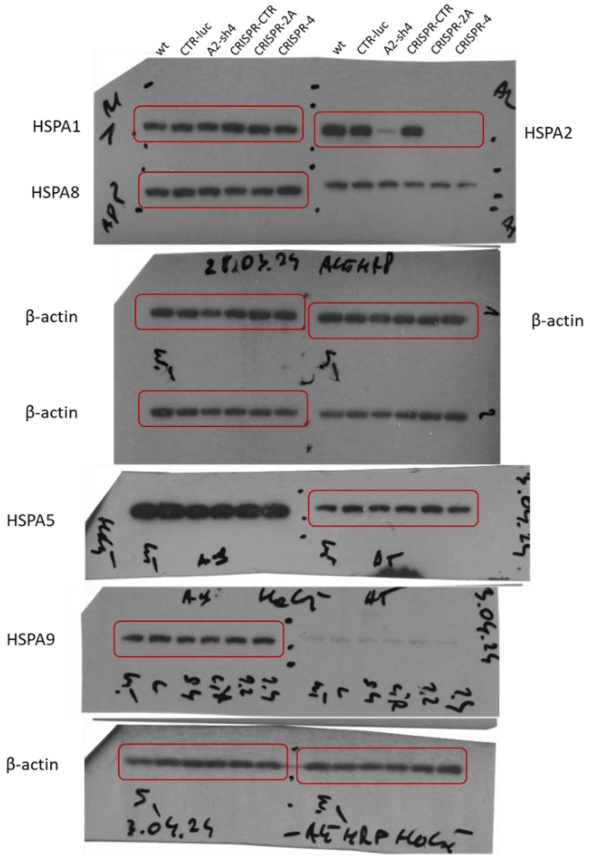

Fig S1B

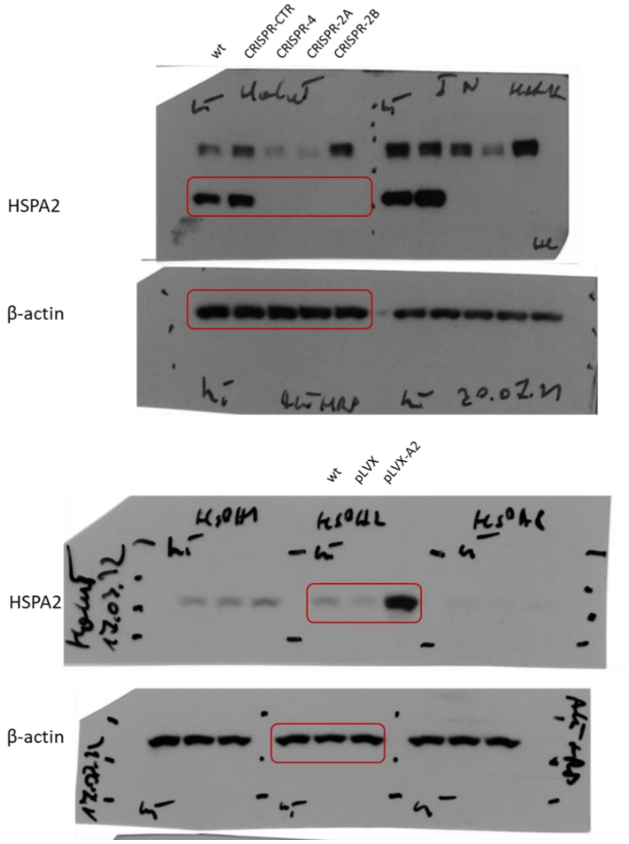

Supplement: Supplementary file 3 — Original data - Uncropped Western Blots [file 41419_2025_7565_MOESM3_ESM.pdf]
